# COMTOP: Protein Residue–Residue Contact Prediction through Mixed Integer Linear Optimization

**DOI:** 10.3390/membranes11070503

**Published:** 2021-06-30

**Authors:** Md. Selim Reza, Huiling Zhang, Md. Tofazzal Hossain, Langxi Jin, Shengzhong Feng, Yanjie Wei

**Affiliations:** 1 School of Computer Science and Technology, University of Chinese Academy of Sciences, Beijing 100049, China; selim@siat.ac.cn (M.S.R.); hl.zhang@siat.ac.cn (H.Z.); tofazzal@siat.ac.cn (M.T.H.); 2 Centre for High Performance Computing, Joint Engineering Research Center for Health Big Data Intelligent Analysis Technology, Shenzhen Institutes of Advanced Technology, Chinese Academy of Sciences, Shenzhen 518055, China; sz.feng@siat.ac.cn; 3 Department of Computer Science and Technology, School of Computer Science and Technology, Harbin University of Science and Technology, 52 Xuefu Road, Nangang District, Harbin 150080, China; 1904010508@stu.hrbust.edu.cn

**Keywords:** protein residue–residue contact, contact prediction, mixed integer linear programming, machine learning, protein sequence

## Abstract

Protein contact prediction helps reconstruct the tertiary structure that greatly determines a protein’s function; therefore, contact prediction from the sequence is an important problem. Recently there has been exciting progress on this problem, but many of the existing methods are still low quality of prediction accuracy. In this paper, we present a new mixed integer linear programming (MILP)-based consensus method: a Consensus scheme based On a Mixed integer linear opTimization method for prOtein contact Prediction (COMTOP). The MILP-based consensus method combines the strengths of seven selected protein contact prediction methods, including CCMpred, EVfold, DeepCov, NNcon, PconsC4, plmDCA, and PSICOV, by optimizing the number of correctly predicted contacts and achieving a better prediction accuracy. The proposed hybrid protein residue–residue contact prediction scheme was tested in four independent test sets. For 239 highly non-redundant proteins, the method showed a prediction accuracy of 59.68%, 70.79%, 78.86%, 89.04%, 94.51%, and 97.35% for top-5L, top-3L, top-2L, top-L, top-L/2, and top-L/5 contacts, respectively. When tested on the CASP13 and CASP14 test sets, the proposed method obtained accuracies of 75.91% and 77.49% for top-L/5 predictions, respectively. COMTOP was further tested on 57 non-redundant α-helical transmembrane proteins and achieved prediction accuracies of 64.34% and 73.91% for top-L/2 and top-L/5 predictions, respectively. For all test datasets, the improvement of COMTOP in accuracy over the seven individual methods increased with the increasing number of predicted contacts. For example, COMTOP performed much better for large number of contact predictions (such as top-5L and top-3L) than for small number of contact predictions such as top-L/2 and top-L/5. The results and analysis demonstrate that COMTOP can significantly improve the performance of the individual methods; therefore, COMTOP is more robust against different types of test sets. COMTOP also showed better/comparable predictions when compared with the state-of-the-art predictors.

## 1. Introduction

Protein contact prediction aims at predicting which residues of a protein are in contact. Two non-local residues are far away from each other in the protein primary structure, but they are close to each other in the 3D structure. Protein contact prediction is helpful in determining protein structure, model ranking, selection, and evaluation [1,2] and is also important for other fields in evolutionary biology and biotechnology, such as protein function prediction and drug design [3]. A protein contact map is a 2D representation of a protein’s 3D structure. Contact map information can be used as distance restraints to guide protein structure modeling [4,5,6,7,8,9,10]. This creates a new direction for solving the grand challenge of the de novo protein structure. The idea of residue–residue contact prediction and using it to predict 3D models was introduced around two decades ago [11,12]; the realization of that idea has only recently gained much attention by the community and has come into practice as many authors have shown how residue contacts can be predicted with reasonable accuracy [13,14,15,16,17,18,19,20].

The last decade has witnessed significant progress in the development of algorithms for protein contact map prediction. The existing residue–residue contact prediction methods can be broadly classified into three categories: (1) coevolution-based, (2) machine-learning-based, and (3) mathematical optimization technique-based. Coevolution information-based analysis predicts contacts by identifying co-evolved residues in a protein, such as plmDCA [13], COLORS [17], EVfold [4], PSICOV [21], CCMpred [15], GREMLIN [22], MetaPSICOV [16], FreeContact [23], and gDCA [24]. These coevolution-based methods use MSA (multiple sequence alignment) as input, which can be generated using methods like PSI-Blast, HHblits, or Jackhmmer. Protein contact map prediction is regarded as a pattern recognition problem in machine-learning-based approaches, and it is solved using machine learning algorithms. Hidden Markov model, support vector machines, and/or artificial neural networks were used in the early development of such methods; falling into this category of methods are FragHMMent [25], SVMcon [26], SVMSEQ [27], and NNcon [28]. In recent years, with the advancement of deep learning techniques, the precision of the predicted contact maps has increased significantly. Methods in this category include PconsC4 [20], DeepCov [19], RaptorX-contact [29], DNCON2 [30], SPOT-contact [31], DeepContact [32], DeepConPred [33], plmConv [34], DeepFold [35], and so on. In the third group of methods, such as recent ones include COMSAT [18], which is based on support vector machines (SVM), and mixed integer linear programming (MILP) for residue–residue contact prediction of TM proteins. Other optimization methods also solved the problem through satisfying categories of constraints in the MILP models to maximize the probability of the sum of residue contacts [36,37,38,39].

By combining multiple methods, prediction accuracy can be improved because non-systematic errors can be removed by correctly combining them. This idea has been broadly used in different areas of computational biology, such as protein contact prediction [16,18,40,41], secondary structure prediction [42,43,44,45], fold recognition and threading [46,47,48,49], protein disorder prediction [50,51,52], prediction of gene coding sequences [53], clustering and functional explanation of gene-expression data [54], computational studies of protein folding [55,56,57,58], etc. One example of consensus methods for protein contact prediction is COMSAT. It is based on support vector machines (SVM) and mixed integer linear programming (MILP) for contact prediction of TM proteins. It is claimed that this combined method overcomes the cross-validation prediction accuracy barrier of 64.5% and a specificity of 99.4% [18]. LOMETS is an online server for protein structure prediction using the consensus method [47]. CONCORD is a consensus method for secondary structure prediction and uses seven selected secondary structure prediction methods. It achieved an average prediction accuracy of about 83.04% with 6-fold cross-validation on a PDBselect25 protein set [44]. Another consensus method for secondary structure prediction is the consensus data mining (CDM) method. It combines two complementary methods having different strengths: GOR and fragment database mining (FDM) methods. It is claimed that this combined method overcomes the cross-validation prediction accuracy barrier of 80% [42]. Kumar and Carugo [50] developed a consensus method for predicting protein conformational disorder with an accuracy of 81.4% based on 12 methods, using least-squares optimization.

Recently exciting progress has been made on this problem, but contact prediction for proteins without many sequence homologs is still of low quality and insufficient for accurate contact-assisted protein folding [59,60]. Jumper [61] recently established AlphaFold2, a novel strategy that used a different deep learning technique than CASP13 AlphaFold to simulate protein 3D structures. However, it had some targets where the prediction was not very good. The protein targets set at the CASP14 conference do not fully represent all the proteins with many unique structural prediction issues. Thus, the algorithm could not be universally applicable to all proteins.

COMTOP is the first consensus method for protein contact prediction using MILP to maximize the probability of the sum of residue contacts, based on the previous work [44]. This method uses seven selected residue–residue contact prediction methods, including CCMpred [15], EVfold [4], DeepCov [19], NNcon [28], PconsC4 [20], plmDCA [13], and PSICOV [21]. COMTOP maximally combines the strengths of seven protein contact prediction methods by optimizing the number of correctly predicted pairs of residues in the training set. A consensus prediction score based on the confidence scores of the seven individual methods is initiated to assess the likelihood of a residue pair being at one of the protein contact states. Our method performed well compared with seven individual methods when tested by 239 proteins, and a prediction accuracy of about 89.04%, 94.51%, and 97.35% for top-L, top-L/2, and top-L/5 predicted contacts, respectively, was obtained. When tested on CASP13, CASP14, and 57 non-redundant TM proteins, the consensus method achieved accuracies of 75.91%, 77.49%, and 73.91% for top-L/5 predictions, which was better than the seven individual methods and could achieve state-of-the-art prediction performance.

## 2. Materials and Methods

### 2.1. Data Description

High-quality training sets and test sets are crucial for the development and validation of prediction models. To train our proposed methods, a training set and a validation set were constructed as follows. We first downloaded a list of 3298 protein chains from PISCES website [62] with a maximum sequence identity of 20%, a maximum R-factor of 0.3, and resolutions better than 2.0 Å. Further, we removed the protein chains with less than 50 amino acids, and 3133 chains (Table S1) were left for our database construction. Then we generated the confidence score by the seven locally installed methods (CCMpred, EVfold, DeepCov, NNcon, PconsC4, PlmDCA, PSICOV) based on 3133 proteins, but some methods could not predict the confidence score for some protein chains. We deleted those proteins from our dataset because our method combines seven individual methods. Following this criterion, we had 1189 proteins in our dataset. The selected protein set was divided into two parts: a training set with 950 proteins (Table S2) and a testing set with 239 proteins (Table S3). Then we ranked the confidence score for the training set and test set, and prepared the dataset for MILP model by selecting the top-5L, top-3L, top-2L, top-L, top-L/2, and top-L/5 predictions where L was the length of a protein.

The second test set was based on the CASP13 protein domains (http://www.predictioncenter.org/download_area/CASP13/, accessed on 5 March 2021). There were 32 target domains, and certain methods were unable to predict the confidence score for two target domains, so they were excluded from the test set, leaving 30 target domains in the CASP13 dataset, which are listed in Table S4 of the Supplementary Materials. 

The third test dataset was based on the CASP14 target domains (https://www.predictioncenter.org/casp14/index.cgi, accessed on 5 March 2021). There were 45 target domains, and 28 of them had PDB IDs. After excluding the targets that were unable to obtain confidence scores from all the seven methods, the CASP14 dataset contained 25 target domains, as shown in Table S5.

Finally, we assessed our method on a non-redundant α-helical TM protein test set consisting of 57 α-helical TM proteins through culling the α-helical TM proteins in the PDBTM database against the training test sets of COMTOP and the individual methods NNcon, DeepCov, and PconsC4 (EVfold, plmDCA, PSICOV, and CCMpred had no training sets) with a maximum sequence identity of 25%, a maximum R-factor of 0.3, and resolutions better than 2.0 Å. The 57 α-helical TM proteins are listed in Table S6 of the Supplementary Materials.

### 2.2. Method Description

#### 2.2.1. List of the Sets, Parameters and Variables

This section lists the sets, parameters, and variables used in this method.

(I) Indices and sets

(*I*,*J*): set of pairs of amino acid positions of a protein, *i*∈*I*, *j*∈*J*;

*P*: set of training proteins, *p*∈*P*;

M: set of the methods used, [1,2,3,4,5,6,7], m∈M. Seven methods were used in this consensus method: m = 1 indicates the CCMpred method; m = 2 indicates the DeepCov method; m = 3 indicates the EVfold method; m = 4 indicates the NNcon method; m = 5 indicates the PconsC4 method; m = 6 indicates the plmDCA method; m = 7 indicates the PSICOV method; and subsetP(*I*,*J*)(*p*,(*I*,*J*)): subset indicates the number of pairs of residues for each protein *p*.

(II) Parameters

*confS*(*p*,(*i*,*j*),*m*): the confidence score predicted by method m for pairs of residues (*i*,*j*) of a protein *p*, *p*∈*P*, (*i*,*j*)∈subsetP(*I*,*J*)(*p*,(*I*,*J*)), m∈M. Firstly the score was normalized for each protein; we used min-max normalization,

(1) $$z_{ij}=\frac{\left.x_{ij}-\min(x_{ij}\right)}{\left.\max\left(x_{ij}\right)-\;\min(x_{ij}\right)}$$

where $x_{ij}$ is the *i*^th^ and *j*^th^ score for a residue pair (*i*,*j*) of a protein. Then we selected the top 4.5 L prediction score from the top 5 L prediction score for each protein and created a score matrix by using top 4.5 L scores of method M; however, the score matrix generated nan-value because all methods were unable to predict the same pair of residues of a protein. Thus, the predictions scoring matrix dealt with missing values by the following strategy;

(2) $$\mathrm{Missing}\;\mathrm{value}\;=\left\{\begin{array}{l} x_{p}-\frac{x_{p}}{\left(y_{p}\;*\;1000\right)},\mathrm{when}\;\mathrm{the}\;\mathrm{pairs}\;\mathrm{is}\;\mathrm{indeed}\;\mathrm{contact}\\ z_{p}\;\;\;\;\;\;,\mathrm{when}\;\mathrm{the}\;\mathrm{pairs}\;\mathrm{is}\;\mathrm{not}\;a\;\mathrm{contact}\; \end{array}\right.$$

where $x_{p}$, $y_{p},$ and $z_{p}$ are the minimum value, maximum value, and average value of the p-th protein. P: set of training proteins, [1…950], *p*∈*P*; predSS(*p*,(*i*,*j*),m): the training model label matrix depends on the real prediction, 1 for a true prediction and 0 for a false prediction, *p*∈*P*, (*i*,*j*)∈subset *P*(*I*,*J*)(*p*,(*I*,*J*)), m∈M. So, we handled the missing nan value by this strategy for label matrix;

(3) $$\mathrm{missing}\;\mathrm{value}\;=\left\{\begin{array}{l} 1\\ 0 \end{array}\begin{array}{c} \mathrm{when}\;\mathrm{the}\;\mathrm{pairs}\;\mathrm{are}\;\mathrm{not}\;a\;\mathrm{contact}\;\\ \;\mathrm{when}\;\mathrm{the}\;\mathrm{pairs}\;\mathrm{are}\;\mathrm{indeed}\;\mathrm{contact}\; \end{array}\right.$$

(III) Binary variables

*y*(*p*, (*i*,*j*)): equals to 1 if the sum of the scores of the correct contact predictions is higher than the sum of the incorrect ones for residue pair (*i*,*j*) of a protein *p* by at least $\epsilon \left(p,\left(i,j\right)\right)$, *p*∈*P*, *i*,*j*∈subset(*I*,*J*) (*P*, (*I*,*J*)); 

y2(p): equals to 1 if the sum of the scores of the correct contact predictions of all pairs of amino acid (*i*,*j*) of a protein *p* is higher than the sum of the score of the incorrect prediction of the same protein *p* by at least $\epsilon 2\left(p\right),$ *p*∈*P*, *i*,*j*∈subset(*I*,*J*) (*P*, (*I*,*J*));

(IV) Positive variables

$\lambda \left(m\right)$: the weight variables for different methods, $0\leq \lambda \left(m\right)\leq 1$, m∈M;

$\epsilon \left(p,\left(i,j\right)\right)$: a soft margin variable for the binary variable y((*p*, (*i*,*j*)), *p*∈*P*, *i*,*j*∈subset(*I*,*J*) (*P*, (*I*,*J*)) (see Section 2.2.1 (III)); and

$\epsilon 2\left(p\right):$ a soft margin variable for the binary variable y2(p), *p*∈*P* (see Section 2.2.1 (III))

#### 2.2.2. The Training Objective Function

For protein contact prediction, the training objective function of the MILP model takes the following format:

(4) $$Max\left\{\underset{\left(p,\left(i,j\right)\right)}{\sum }\left.y(p,\left(i,j\right)\right)-\underset{\left(p,\left(i,j\right)\right)}{\sum }\epsilon \left(p,\left(i,j\right)\right)\right\},\forall \;\left(p,\left(i,j\right)\right)\in subsetP\left(I,J\right)\left(P,\;\left(I,J\right)\right)$$

where *y*(*p*,(*i*,*j*)) is a set of binary variables, and it equals to 1 if the sum of the scores of the correct contact predictions is higher than the sum of the incorrect ones for pairs of amino acids (*i*,*j*) of a protein *p* by at least *ϵ*(*p*,(*i*,*j*)), and this objective function is to maximize the total number of pair of residues. $\epsilon \left(p,\left(i,j\right)\right)$ is included here to minimize the sum of soft margins. 

The training objective function was conducted on the individual contact/pairs of residues of a protein. The principle of this function is that some protein contact prediction approaches have better prediction performance in some native regions of a protein than other approaches. The consensus approach aims to identify the correct contact prediction for proteins from various approaches by relying on confidence scores for each contact of residues in a protein.

#### 2.2.3. The Model Constraints

For the protein contact prediction, there are two basic constraints in the consensus scheme. The first constraint makes sure that the binary variable *y*(*p*,(*i*,*j*)) is equal to zero for each contact of residues of a protein if the difference between the sum of the scores of correct contact predictions and the sum of the scores of incorrect predictions from different methods is lower than *e*(*p*,(*i*,*j*)); this constraint is expressed as:

(5) $$\begin{array}{c} \underset{m}{\sum }\lambda \left(m\right)\;*\;confS\left(p,\left(i,j\right),m\right)\;*\;\left(1-predSS\left(p,\;\left(i,j\right),m\right)\right)-\underset{m}{\sum }\lambda \left(m\right)\;*\;confS\left(p,\left(i,j\right),m\right)\;*\;predSS\left(p,\left(i,j\right),m\right)\\ +\epsilon \left(p,\left(i,j\right)\right)<1-y\left(p,\;\left(i,j\right)\right),\forall \;\left(p,\;\left(i,j\right)\right)\in subsetP\left(I,J\right)\left(P,\left(I,J\right)\right),\;m\in M \end{array}$$

The second type of constraint used in the model normalizes the weights terms $\lambda \left(m\right)$ of the seven methods.

(6) $$\underset{m}{\sum }\lambda \left(m\right)=1,\lambda \left(m\right)\geq 0,\;m\in M$$

#### 2.2.4. The Prediction Score and Prediction Label

By carefully selecting the weight variables $\lambda \left(m\right)$ of each individual method through the MILP optimization-based approach, the consensus method was developed to score higher for the correct contact predictions than for the incorrect contact predictions from different methods. The consensus method ensures that pairs of residues have contact if the sum of scores of correct predictions is higher than the sum of scores of incorrect predictions of each pairs of residues for different methods. It is expressed as

(7) $$S\left(p,\left(i,j\right),\;m\right)\;=\;\underset{m}{\sum }\lambda \left(m\right)\;*\;confS\left(p,\left(i,j\right),m\right)\;,\;\;\;\;\;\forall \;\left(p,\;\left(i,j\right)\right)\in subsetP\left(I,J\right)\left(P,\left(I,J\right)\right),\;m\in M$$

where *S*(*p*,(*i*,*j*),*m*) is the consensus contact confidence score for (*i*,*j*) residues of *p*th protein and *m*th method, *conf*(*p*,(*i*,*j*), *m*) is the confidence score for the *m*th method, λ(*m*) is the weighting factor for the *m*th method.

The consensus label matrix is the prediction result of method m for the (*i*,*j*) residues of a protein *p* (a value of 1 corresponds to a true prediction, a value of 0 corresponds to a false prediction). It is expressed by

(8) $$\mathrm{Label}\;\mathrm{matrix}=\left\{\begin{array}{l} 1,\;\mathrm{returned}\;\mathrm{prediction}\;\mathrm{by}\;\mathrm{methods}\;\\ 0,\;\mathrm{not}\;\mathrm{returned}\;\mathrm{prediction}\;\mathrm{by}\;\mathrm{methods} \end{array}\right.$$

#### 2.2.5. The Training and Prediction Procedure

The MILP based training system uses CPLEX (ILOG CPLEX 8.0 reference manual) to optimize the training objective function, from which the weight parameters $\lambda \left(m\right)$ are attained. The training system for each fold takes around two/four weeks. For the prediction procedure, the seven individual programs run in parallel rather than serially. Once we get the results from the individual methods, the prediction from the MILP model of COMTOP can finish in one second. The running time taken by COMTOP depends on the slowest time of the seven methods, and each protein contact prediction takes around 5–25 min, depending on the size of the database used for sequence-profile/MSA analysis.

#### 2.2.6. Evaluation Measures for Prediction Performance

The effectiveness of our proposed method was evaluated by five widely used metrics: the prediction accuracy, coverage, specificity, negative predictive value (NPV), and Matthews’s correlation coefficient (MCC).

The accuracy is defined as the ratio of correct predictions to total predictions. Accuracy can also be written in terms of true positives (TP) and false positives (FP), as shown in Equation (9). A higher value of accuracy means a better contact prediction model.

(9) $$\mathrm{Accuracy}=\frac{N_{\mathrm{corr}}}{N_{\mathrm{pred}}}=\frac{\mathrm{TP}}{\mathrm{TP}+\mathrm{FP}}$$

where $N_{\mathrm{corr}}$ is the number of correctly predicted protein contacts, $N_{\mathrm{pred}}$ is the number of total predicted contacts, TP is the number of true positive contacts, and FP is the number of false positive contacts.

Coverage, also called the true positive rate or referred to as “sensitivity” is defined as the ratio of correct predictions to the number of protein contacts in the native structure, as shown in Equation (10). A higher value of coverage means a better contact prediction model.

(10) $$\mathrm{Coverage}=\frac{N_{\mathrm{corr}}}{N_{\mathrm{native}}}=\frac{\mathrm{TP}}{\mathrm{TP}+\mathrm{FN}}$$

where, $N_{\mathrm{corr}}$ is the number of correctly predicted protein contacts, $N_{\mathrm{native}}\;$ is the number of protein contacts in the native structure, and FN is the number of false negative contacts.

Specificity, also called the true negative rate, is the percentage of predicted contacts that are present in the native structure, as shown in Equation (11). It denotes how good the test is at identifying negative conditions.

(11) $$\mathrm{Specificity}=\frac{\mathrm{TN}}{\mathrm{TN}+\mathrm{FP}}$$

where TN is the number of true negative contacts.

When a screening test returns a negative result, the negative predictive value (NPV) is the probability of properly detecting all pairs of residues that do not have contact from among pairs of residues that might or might not have contact and can be calculated using the following formula:

(12) $$\mathrm{NPV}=\frac{\mathrm{TN}}{\left(\mathrm{TN}+\mathrm{FN}\right)}$$

The last metric used to measure the performance of the contact prediction method was Matthew’s correlation coefficient (MCC), a measure of the quality of two-class classifications, which can be calculated using the following formula:

(13) $$\mathrm{MCC}=\frac{\left(\mathrm{TP}\times \mathrm{TN}\right)-\left(\mathrm{FP}\times \mathrm{FN}\right)}{\sqrt{\left(\mathrm{TP}+\mathrm{FP}\right)\;*\;\left(\mathrm{TP}+\mathrm{FN}\right)\;*\;\left(\mathrm{TN}+\mathrm{FP}\right)\;*\;\left(\mathrm{TN}+\mathrm{FN}\right)}}$$

The proposed method was developed with the purpose of producing higher accuracy, so we consider that accuracy should have a higher weight than the other metrics.

## 3. Results

### 3.1. Performance Evaluation Based on the Training Set

**Figure 1:**
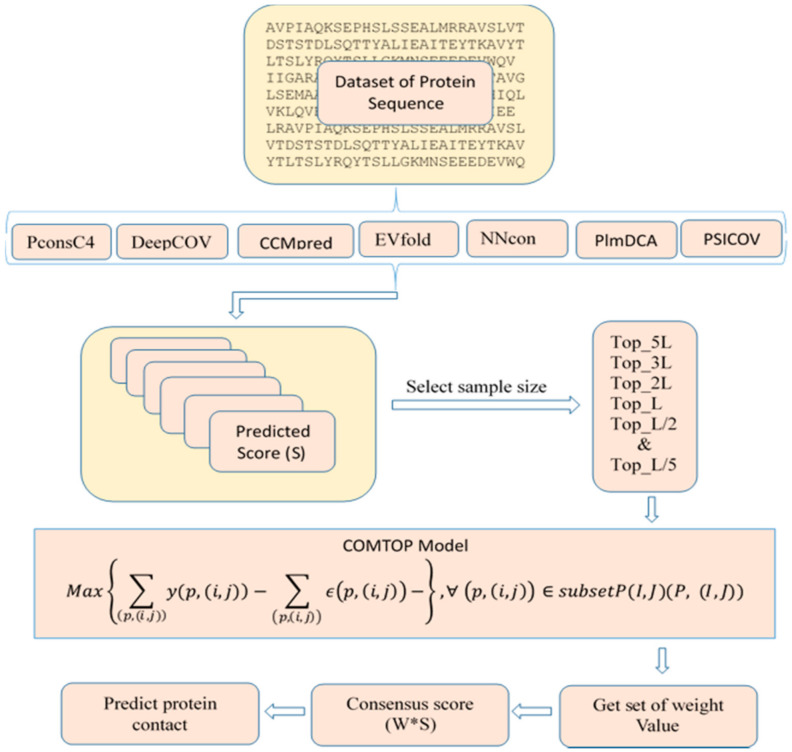
The workflow of COMTOP model for protein contact prediction.

The overall workflow of protein contact prediction is illustrated in Figure 1. The measure of COMTOP performance depends on the weight values given by seven individual methods, which balances the accuracy, coverage, specificity, and MCC. For the training process, a set of optimal parameters from the MILP model were generated (listed in Supplementary Table S7); these parameters were the weights for the seven individual methods that should be used in the consensus prediction model.

**Figure 2:**
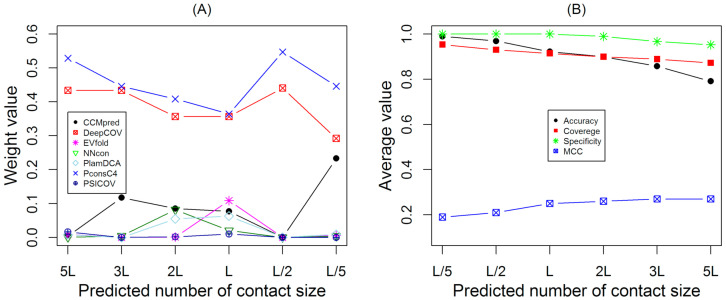
The weight values and the overall performance for the training set. (**A**) The weight values of PconsC4, DeepCov, CCMpred, EVfold, plmDCA, PSICOV, and NNcon with the variation of the sample size. (**B**) The average accuracy, coverage, specificity, and MCC of COMTOP at different sample sizes.

Figure 2 shows the weight values and overall performance with different numbers of sample sizes for the training dataset. Figure 2A shows that PconsC4 generated the highest weight value and DeepCov generated the second highest weight value, while the PSICOV and NNcon generated the lowest weight values at different sample sizes. The fact that different systems reveal different weights is owing to the different prediction accuracies of each method. Similarity between seven different approaches plays an important role in determining the weight values (Table 1). Jaccard’s similarity coefficient value was 0.585 between PconsC4 and DeepCov, so PconsC4 and DeepCov generated the highest weight values. CCMpred, PSICOV, and EVfold are similar type methods (see Table 1) and generated lowest weight values. This explains why CCMpred, plmDCA, NNcon, and PISICOV methods had the smallest weights. On the other hand, note that even though the CCMpred method has the third highest accuracy after PconsC4 and DeepCov for contact prediction, its weight for two cases was very small. This is because many of these seven contact prediction methods use PSI-Blast, HHblits, or Jackhmmer (e.g., CCMpred, EVfold, plmDCA, and PISICOV), artificial neural networks use NNcon and deep learning approaches (e.g., DeepCov, PconsC4), and the prediction results of the different methods may correlate with each other in some fashion. 

Figure 2B shows overall performance based on the training dataset, the average accuracies, coverages, specificities, and MCCs for COMTOP are plotted against different sample sizes. The prediction accuracy, coverages, and specificities were highest when the sample sizes were small, such as top-L/5 and top-L/2 predictions, respectively, while the accuracy and coverage decreased monotonically with the increasing sample size. This represents the classic trade-off phenomenon common to many prediction problems. Although MCC is also an important estimator in protein contact prediction evaluation, this value increased with increasing sample size. The prediction accuracy of the different training models based on the different sample size was 98.99%, 96.88%, 92.18%, 89.91%, 85.77%, and 79.09%, respectively.

### 3.2. Performance Evaluation Based on the Independent Set

**Figure 3:**
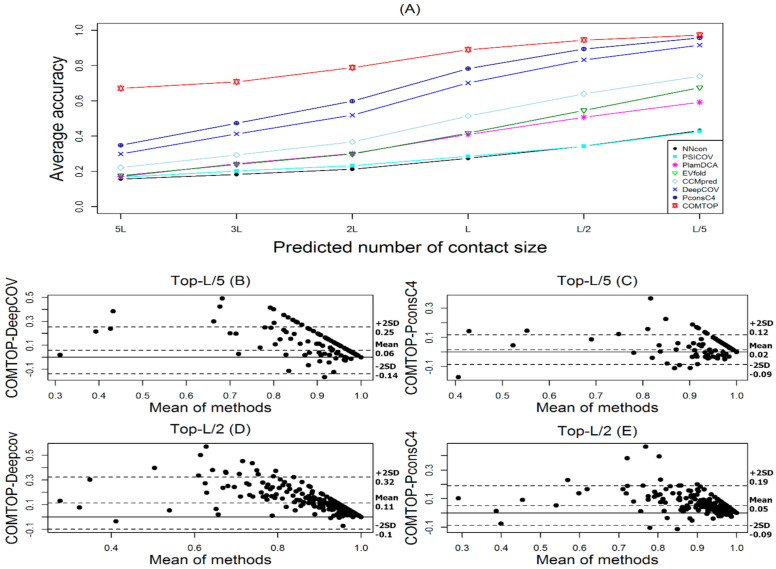
The overall performance of the COMTOP model. (**A**): the prediction accuracy of COMTOP with seven individual methods against predicted number of protein contacts for 239 proteins test dataset. (**B**,**D**): Bland–Altman plots between COMTOP and DeepCov, with the representation of the limits of agreement (dotted line), from +2SD to −2SD. (**C**,**E**): Bland–Altman plots between COMTOP and PconsC4, with the representation of the limits of agreement (dotted line), from +2SD to −2SD.

**Figure 4:**
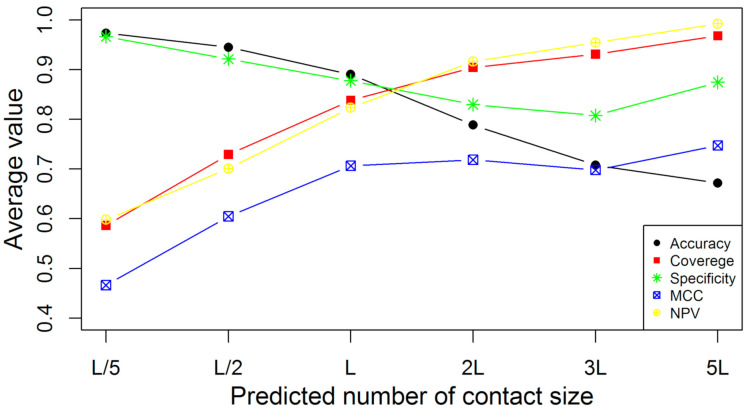
The average accuracy, coverage, specificity, NPV, and MCC of COMTOP in terms of the predicted contact size on the 239 non-redundant proteins.

The measure of COMTOP’s performance for residue contact prediction depends on the best range that balances the accuracy, coverage, specificity, NPV, and MCC. We used four datasets to evaluate the performance of our method (see Section 2.1). Figure 3 and Figure 4 and Table 2 summarize the performance of COMTOP in terms of accuracy (alternatively known as positive predictive value) compared with the seven individual methods when applied to the 239 test proteins. 

We have evaluated the accuracy of the top L/k (k = 5, 2, 1) and top K*L (K = 5, 3, 2, 1) predicted contacts where L is the length of a protein. The prediction accuracy is defined as the percentage of native contacts among the top L/k and KL predicted contacts. On 239 proteins, we evaluated our method using the suggested weight values. The prediction results of our method, together with the other seven individual methods, are shown in Table 2 and Figure 3. We observe that our method generated the highest prediction scores compared with the seven individual methods. 

The COMTOP model has six sub-models (see in Table S7), and the accuracy of the COMTOP model was 59.68%, 70.79%, 78.86%, 89.04%, 94.51%, and 97.35% for top-5L, top-3L, top-2L, top-L, top-L/2, and top-L/5 predictions, respectively. Among the seven methods, the PconsC4 method had the highest score of accuracy with 34.79%, 47.35%, 59.85%, 78.29%, 89.41%, and 95.76% for top-5L, top-3L, top-2L, top-L, top-L/2, and top-L/5 predictions, respectively. The DeepCov method had the second highest score of accuracy with 29.89%, 41.27%, 51.92%, 70.21%, 83.28%, and 91.61% for top-5L, top-3L, top-2L, top-L, top-L/2, and top-L/5 predictions, respectively. On the other hand, the NNcon method had the lowest score of accuracy with 15.66%, 18.27%, 21.24%, 27.44%, 34.34%, and 43.18% for top-5L, top-3L, top-2L, top-L, top-L/2, and top-L/5 predictions, respectively. The prediction accuracies of all the methods were ranked as follows: PconsC4 > DeepCov > CCMpred > EVfold > plmDCA > PSICOV > NNcon, whereas the ranking of all methods by weight values was as follows: PconsC4 > DeepCov > CCMpred > plmDCA > EVfold > NNcon > PSICOV. The weight values are highly dependent on an individual method’s performance. The improvement of COMTOP in accuracy over the seven individual methods increased with the increasing number of predicted contacts. For example, the improvements of COMTOP in accuracy over the best individual method PconsC4 were 24.89%, 23.34%, 19.01%, 10.75%, 5.1%, and 1.59% for top-5L, top-3L, top-2L, top-L, top-L/2, and top-L/5 predictions, respectively.

Figure 3A shows the overall performance of the COMTOP model in terms of its average accuracy. For comparison, results for CCMpred, EVfold, DeepCov, NNcon, PconsC4, PlmDCA, and PSICOV are also shown. Clearly, the COMTOP model significantly outperforms the other individual methods. To see this in more detail, Figure 3B–E shows the Bland–Altman plot indicating the relationship of COMTOP against PconsC4 and DeepCov. For Figure 3B–E, the majority of the points are above the zero line, and about 95%, 95.8%, 95.4%, and 97.5% fall within the confidence limit, respectively. The mean/bias values of the differences are also all positive, indicating that COMTOP outperformed DeepCov and PconsC4 for test datasets [63].

In Figure 4, the average accuracies, coverages, specificities, NPVs, and MCCs for COMTOP are plotted against the different sample sizes on the 239 non-redundant proteins. The prediction accuracies and specificities are highest when the sample sizes are small, such as top-L/5 predictions, while the accuracy and specificities decrease monotonically with the increasing sample size. The accuracies of the COMTOP model for top-5L, top-3L, top-2L, top-L, top-L/2, and top-L/5 predictions were 59.68%, 70.79%, 78.86%, 89.04%, 94.51%, and 97.35%, respectively, while the corresponding NPVs were 99.22%, 95.41%, 91.66%, 82.34%, 70.10%, and 59.85%, respectively [64]. The coverage improves monotonically with the increase of the sample size. This represents the classic trade-off phenomenon common to many prediction problems. Although MCC is also an important estimator in protein contact prediction evaluation, this value is highest when the sample size is top-L and top-2L. More importantly, top-L is the best range for protein contact prediction. Concerning the number of contacts required for accurate folding, the top-L contacts have been shown to produce good results [65,66]; nevertheless, the researchers have recommended that the number of contacts required be specific to the prediction methods.

### 3.3. Testing on CASP13 Targets

The critical assessment of protein structure prediction (CASP) is a biennial worldwide competition for protein structure prediction, identifying what progress has been made and highlighting where future effort may be most productively focused. The competition unfolds in a double-blind fashion: The structures of the target domains are unknown to the predictors and the organizers (http://predictioncenter.org/download_area/CASP13/, accessed on 25 April 2021).

#### 3.3.1. Comparison of COMTOP’s Performance with the Seven Individual Methods

**Figure 5:**
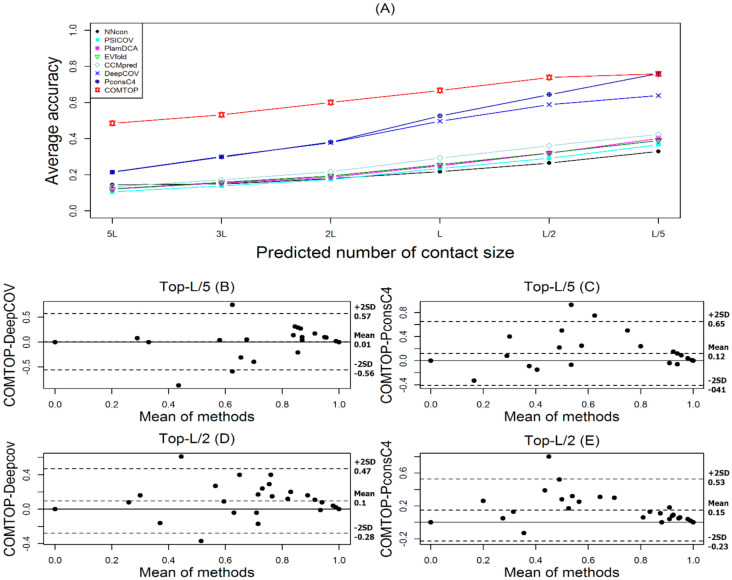
The overall performance of the COMTOP model. (**A**): the prediction accuracy of COMTOP with seven individual methods against predicted number of protein contacts size for CASP13 dataset. (**B**,**D**): Bland–Altman plots between COMTOP and DeepCov, with the representation of the limits of agreement (dotted line), from +2SD to −2SD. (**C**,**E**): Bland–Altman plots between COMTOP and PconsC4, with the representation of the limits of agreement (dotted line), from +2SD to −2SD.

In this section, we tested COMTOP on 30 domains in CASP13 using the proposed parameter sets listed in Table S7. The prediction results of COMTOP, together with the other seven individual methods, are shown in Table 3 and Figure 5A, and the COMTOP model significantly beat the other individual techniques. As shown in Table 3, the improvement of COMTOP in accuracy over the seven individual methods increases with the increasing number of predicted contacts. For example, the improvement of COMTOP in accuracies over the top individual method DeepCov were 27.1%, 23.40%, 22.0%, 14.1%, 9.5%, and 0.81% for top-5L, top-3L, top-2L, top-L, top-L/2, and top-L/5 predictions, respectively. 

**Figure 6:**
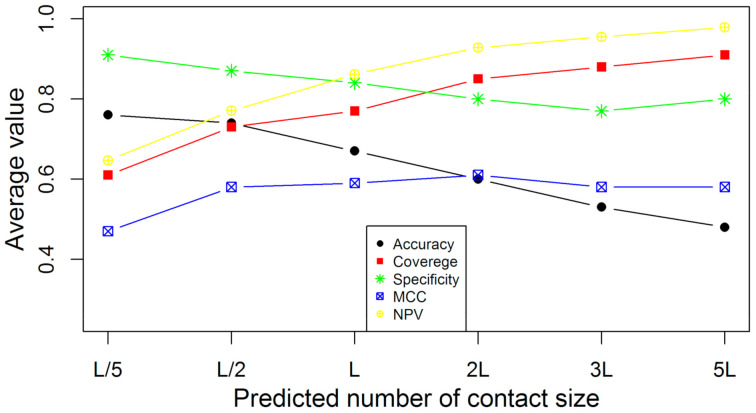
The average accuracy, coverage, specificity, NPV, and MCC of COMTOP at predicted contact sizes on CASP13 dataset.

Figure 5B–E as Bland–Altman plots shows the relationship of COMTOP against PconsC4 and DeepCov. For Figure 5B–E, the majority of the points are above the zero line, and about 93.4%, 93.4%, 93.4%, and 96.7% fall within the confidence limit, respectively. The mean/bias of the differences are also shown to be all positive, indicating that COMTOP outperformed DeepCov and PconsC4 for CASP13 datasets. In Figure 6, the average accuracies, coverages, specificities, NPVs, and MCCs for COMTOP are plotted against the different number of contact predictions for 30 domains on CASP13 datasets. For top-L/5 predictions, the accuracy and specificity of the COMTOP model were 75.91% and 91.24%, respectively, while NPVs and coverage were 64.66% and 61.45%. Although MCC is also an important estimator in protein contact prediction evaluation, this value was highest when the sample size was top-5L and top-2L, respectively. 

Table 4 shows the accuracies achieved by COMTOP on the domains classified as FM/TBM-easy/TBM-hard/FM/TBM based on CASP13 datasets. Over these target domains, COMTOP achieved average accuracies of 75.91% and 73.90% when considering the top-L/5 and top-L/2 predictions. For top-L/5 predictions, COMTOP showed prediction accuracies larger than 90% for 18 domains and accuracies of about 100% for 16 of these domains. Remarkably, our system obtained high accuracies for TBM-easy, TBM-hard, and FM/TBM classification domain. Among all the individual methods, DeepCov performed best with an accuracy value of 21.40%, 29.80%, 38.10%, 52.60%, 64.40%, and 75.10% for top-5L, top-3L, top-2L, top-L, top-L/2, and top-L/5 predictions, respectively. CCMpred, EVfold, and plmDCA methods had the same ranking as the test set, with 239 proteins, but DeepCov, PconsC4, PSICOV, and NNcon showed different rankings. The prediction accuracies of all the methods were ranked as follows: DeepCov > PconsC4 > CCMpred > EVfold > plmDCA > NNcon > PSICOV, whereas the ranks of all methods based on the test set with 239 proteins were as follows: PconsC4 > DeepCov > CCMpred > EVfold > plmDCA > PSICOV > NNcon.

The prediction accuracy of COMTOP was reduced from an accuracy of 97.35% on the 239 proteins to 75.91% on the CASP13 dataset for the top-L/5 predictions. Moreover, the prediction accuracy of COMTOP was reduced about 11.18%, 17.59%, 18.76%, 22.34%, 20.61%, and 21.45% in CASP13 for top-5L, top-3L, top-2L, top-L, top-L/2, and top-L/5 predictions, respectively. Among the seven individual methods, PconsC4 method had the largest decrease in accuracy, about 13.29%, 17.25%, 29.75%, 28.59%, 30.51%, and 31.86% in CASP13 for top-5L, top-3L, top-2L, top-L, top-L/2, and top-L/5 predictions, respectively. The CCMpred method had the second largest decrease in accuracy, about 8.91%, 12.14%, 19.56%, 22.13%, 27.88%, and 31.70% in CASP13 for top-5L, top-3L, top-2L, top-L, top-L/2, and top-L/5 predictions, respectively. On the other hand, PSICOV performed better for CASP13 targets than the test dataset only for top-L/5 predicted contacts; the prediction accuracy improved from 40.45% to 42.06%.

#### 3.3.2. Comparison of COMTOP’s Performance with a Few State-of-the-Art Schemes

Finally, we compared our COMTOP system with a few state-of-the-art systems that were not used for developing COMTOP; another set of five contact prediction systems was chosen: RapterX [29], Yang_Server [67], TripletRes [68], ResTriplet [68], and DNCON3 [69]. For this assessment, 20 domains were selected from the CASP13 dataset for which the native structures were publicly available, and all 7 methods generated the results. These 20 domains included both FM (free-modeling) and TBM (template-based modeling) domains. To get the predicted contacts for other methods, we evaluated the contact predictions of the top-L/2 and top-L/5 groups in CASP13 over these 20 targets from the webserver at https://predictioncenter.org/casp13/rrc_results.cgi, accessed on 25 April 2021.

The performance of the COMTOP scheme, together with five state-of-the-art systems in terms of accuracy of top-L/2 and top-L/5 predictions is shown in Table 5. From the table, we can see that COMTOP performed better than Yang_server, TripletRes, ResTriplet, and DNCON3 schemes for the top-L/2 contacts and better than TripletRes, ResTriplet, and DNCON3 schemes for the top-L/5 predictions. On the other hand, our model had accuracies of 84.02% and 88.87% for top-L/2 and top-L/5 predictions, respectively, after RapterX. In addition, Yang_server has a slightly better performance than our method for top-L/5 predictions.

### 3.4. Testing on CASP14 Targets

In this section, we tested COMTOP on 25 CASP14 target domains using the proposed parameter sets. These 25 domains in the CASP14 dataset included both FM and TBM domains, for which the native structures were publicly available.

#### 3.4.1. Performance Comparison of COMTOP against the Seven Individual Methods

Table 6 shows the COMTOP prediction results alongside the seven individual methods, and the COMTOP model significantly outperformed the other methods.

For top-L/2 and top-L/5 predictions, COMTOP achieved average accuracies of 68.33% and 77.49%, respectively. Our prediction accuracies were more than 90% for 13 domains, with 11 of these domains showing 100% accuracies for top-L/5 predictions. Among all the individual methods, DeepCov performed best with accuracies of 67.65% and 71.86% for top_L/2 and top_L/5 contacts, respectively. The overall ranking differed from the CASP13 dataset. DeepCov, PconsC4, PSICOV, and plmDCA methods had the same ranking as the CASP14 dataset, but the ranking of EVfold, NNcon, and CCMpred changed. The prediction accuracies of all the methods were ranked as follows: DeepCov > PconsC4 > NNcon > CCMpred > plmDCA > EVfold > PSICOV. As shown in Table 6, the improvement of COMTOP in accuracy over the seven individual methods increased with the increasing number of predicted contacts. 

For the CASP14 dataset, COMTOP’s prediction accuracy for top-L/5 contacts increased by 1.58% compared with the CASP13 dataset; overall it reduced by 7.23%, 6.28%, 7%, 5.52%, and 5.57% for the top-5L, top-3L, top-2L, top-L, and top-L/2 predictions. In addition, COMTOP’s prediction accuracy for the top-L/2, and top-L/5 contacts decreased by 26.18% and 19.86% in the CASP14 dataset from the 239 proteins test dataset. Other methods also showed a decreasing trend in accuracy for CASP14 than for CASP13 and the 239 proteins test dataset, indicating that CASP14 was more difficult than others. Among the seven individual methods, the best performing method, DeepCov, decreased in accuracy for top-L/5 contacts, approximately by 3.24% and 19.75% in the CASP14 from the CASP13 and the test set with 239 proteins, respectively. The second-best method, PconsC4, showed a 5.56% improvement in accuracy for top-L/5 contacts in the CASP14 from CASP13 dataset but a 26.3% decrease from the 239 proteins test dataset.

#### 3.4.2. Performance Comparison of COMTOP against State-of-the-Art Methods

We compared our COMTOP model against a group of seven state-of-the-art contact prediction systems that were recently developed: MULTICOM-AI [70], Kiharalab_Contact [71], tFold, DeepPotential, trfold, RapterX [29], and TripletRes [68]. To get the prediction accuracy for state-of-the-art systems, we took the contact predictions of the top-L/2 and top-L/5 predictions of these methods from the webserver at https://predictioncenter.org/casp14/rrc_results.cgi, accessed on 25 April 2021. Table 7 compares the prediction accuracy of the COMTOP method with state-of-the-art systems on the CASP14 dataset. From Table 7, COMTOP performed better than tFold, MULTICOM-AI, RaptorX, trfold, and Kiharalab_Contact schemes for the top-L/2 and top-L/5 predictions, respectively. On the other hand, TripletRes and DeepPotential had better performance for top-L/2 and top-L/5 predictions. 

### 3.5. Testing on the Independent TM Test Set

In this section, we tested COMTOP on 57 α-helical TM proteins using the proposed parameter sets. Table 8 compares the COMTOP prediction results to the seven individual approaches, revealing that the COMTOP model beat the others by a substantial margin. COMTOP obtained an average accuracy of 41.56%, 53.38%, 64.34%, and 73.91% for the top-2L, top-L, top-L/2, and top-L/5 predictions, respectively.

Among the seven methods, PconsC4 had the highest accuracy with 37.32%, 45.44%, 55.16%, and 65.26% for top-2L, top-L, top-L/2, and top-L/5 predictions, respectively. The DeepCov method had second-highest score of accuracy with 35.45%, 42.34%, 53.24%, and 63.03% for top-2L, top-L, top-L/2, and top-L/5 predictions, respectively. On the other hand, the PSICOV method had the lowest accuracy with 24.75%, 25.21%, 26.83%, and 29.17% for top-2L, top-L, top-L/2, and top-L/5 predictions, respectively. The prediction accuracies of all the methods were ranked as follows: PconsC4 > DeepCov > CCMpred > plmDCA > EVfold > NNcon > PSICOV, whereas the ranking of all methods based on test dataset was as follows: PconsC4 > DeepCov > CCMpred > EVfold > plmDCA > PSICOV > NNcon. COMTOP’s prediction accuracy for top-L/5 contacts decreased by 23.44%, 2%, and 3.58% in the TM protein dataset from the 239 non-redundant proteins, CAS13, and CASP14 datasets, respectively. 

## 4. Discussion

In this paper, the proposed hybrid framework COMTOP model used information from the seven individual methods that were different from each other in terms of both methodology and input features. The seven methods can be roughly classified into three different categories: traditional machine learning, evolutionary coupling analysis, and deep learning. These methods also rely on different input data types. As shown in our previous work [72], the prediction results of these methods show certain degrees of similarity and difference, and the differences of prediction results from methods in the different categories are larger than that in the same category. COMTOP selects the weight variables for each individual method through the MILP optimization-based approach. Our consensus method scores higher for the correct contact predictions than for the incorrect contact predictions from the different methods; thus, individual methods with higher accuracy usually obtain higher weights. The seven methods can complement each other in prediction performance, so our method produces higher accuracy compared with seven individual methods and shows better or close prediction performance when compared with other state-of-the-art methods.

These seven methods were selected among the available methods based on two criteria: methods that (1) have comparatively better prediction accuracy and (2) belong to different method categories and complement each other. These seven contact prediction methods are classified into two categories: (1) coevolution-derived information-based, and (2) machine-learning-based. Most of these coevolution-derived approaches have been used MSA as input, which can be generated by approaches such as PSI-Blast, HHblits, or Jackhmmer. Most of these machine-learning-based methods have accepted a wide range of features as input, including features involved with the local window of the amino acids, amino acid type information (polarity and acidic properties), and the protein itself. This includes features such as mutual information of sequence profiles, information about the amino acid type (polarity and acidic properties), sequence profiles, sequence separation length between the amino acids under consideration, secondary structure, solvent accessibility, and pairwise information between all the amino acids involved [73]. The commonly used machine learning techniques for contact prediction are hidden Markov model, support vector machines, shallow neural networks and deep learning techniques.

COMTOP uses the confidence scores of the predicted contact and the weights of the seven individual methods to determine the protein residue–residue contact. The contact prediction of COMTOP is based on the sum of the products between the confidence score and the weight term over all methods; thus, the consensus method can also provide the confidence scores of the prediction for each pair of residues. Although the results for COMTOP use seven individual methods, COMTOP consistently shows better performance than the seven individual methods for top-5L, top-3L, top-2L, top-L, top-L/2, and top-L/5 contacts. It can be seen in Table 2 that the prediction accuracy of our methods is about 24.89%, 23.44%, 19.01%, 10.75%, 5.10%, and 1.59% better than the best of the individual methods, PconsC4, for top-5L, top-3L, top-2L, top-L, top-L/2, and top-L/5 contacts, respectively. 

We also tested the performance of our method on CASP13, CASP14, and a non-redundant TM protein test set. The prediction accuracies were 75.91%, 77.49%, and 73.91%, respectively, for top-L/5 contacts. The prediction accuracies of COMTOP for all test sets significantly outperformed those of the seven individual methods. Furthermore, when we compared our method with a few state-of-the-art methods, it can be observed that the RapterX method showed better performance than our model for the CASP13 dataset. The COMTOP model outperformed Yang_server, TripletRes, ResTriplet, and DNCON3 schemes for the top-L/2 and top-L/5 contact predictions for CASP13 targets. In the CASP14 dataset, COMTOP performs better than tFold, MULTICOM-AI, RaptorX, trfold, and Kiharalab_Contact schemes for the top-L/2 and top-L/5 contacts, respectively. On the other hand, TripletRes and DeepPotential have better performance for top-L/2 and top-L/5 contacts. The results and analysis demonstrate that COMTOP can significantly improve the performance of the individual methods; therefore, COMTOP is more robust against different types of test sets. COMTOP also showed better/comparable predictions when compared with the state-of-the-art predictors.

## 5. Conclusions

In this paper, we presented a novel hybrid consensus method named as COMTOP and based on seven methods, aiming to predict high-quality protein contacts that can be used for 3D structure prediction. This consensus contact prediction method is based on a MILP model that produces the parameters for protein residue–residue contact prediction. The test on the 239 targets showed that COMTOP performed well compared with seven individual methods according to the prediction accuracy. COMTOP achieved a prediction accuracy of 75.91%, 77.49%, and 73.91% for top-L/5 contacts test on the CASP13, CASP14, and 50 TM target proteins, respectively, and showed satisfactory results compared with the state-of-the-art predictors. For all test datasets, the improvement of COMTOP in accuracy over the seven individual methods increases with the increasing number of predicted contacts. For example, COMTOP performs much better with a large number of contact predictions (such as top-5L and top-3L) than for a small number of contact predictions, such as top-L/2 and top-L/5.

## Figures and Tables

**Table 1:** Jaccard’s similarity coefficient based on training dataset of seven methods.

	NNcon	EVfold	plmDCA	PSICOV	CCMpred	DeepCov	PconsC4
Nncon	1						
EVfold	0.076	1					
plmDCA	0.064	0.353	1				
PSICOV	0.068	0.488	0.351	1			
CCMpred	0.079	0.517	0.306	0.540	1		
DeepCov	0.210	0.215	0.176	0.243	0.285	1	
PconsC4	0.194	0.291	0.207	0.321	0.375	0.585	1

**Table 2:** The prediction accuracies for COMTOP and the seven individual methods (PconsC4, DeepCov, CCMpred, EVfold, plmDCA, PSICOV, and NNcon) on the 239 non-redundant proteins.

Methods	Top-5L	Top-3L	Top-2L	Top-L	Top-L/2	Top-L/5
NNcon	15.66	18.27	21.24	27.44	34.34	43.18
PSICOV	16.75	20.26	23.28	28.60	34.26	42.71
plmDCA	17.11	24.39	30.16	40.98	50.72	59.26
EVfold	17.58	24.04	29.89	41.80	54.62	67.57
CCMpred	22.21	29.34	36.76	51.43	63.98	74.00
DeepCov	29.89	41.27	51.92	70.21	83.28	91.61
PconsC4	34.79	47.35	59.85	78.29	89.41	95.76
**COMTOP**	**59.68**	**70.79**	**78.86**	**89.04**	**94.51**	**97.35**

**Table 3:** The prediction accuracy on CASP13 target domains for COMTOP with seven individual methods (PconsC4, DeepCov, CCMpred, EVfold, plmDCA, PSICOV, and NNcon).

Methods	Top-5L	Top-3L	Top-2L	Top-L	Top-L/2	Top-L/5
PSICOV	14.50	14.90	17.90	21.80	26.50	32.90
NNcon	10.50	13.90	17.40	23.50	29.10	36.70
plmDCA	12.10	15.40	18.70	25.00	32.00	40.10
EVfold	12.20	15.80	19.30	25.60	32.10	39.00
CCMpred	13.30	17.20	21.70	29.30	36.10	42.30
PconsC4	21.50	30.10	37.80	49.70	58.90	63.90
DeepCov	21.40	29.80	38.10	52.60	64.40	75.10
**COMTOP**	**48.50**	**53.20**	**60.10**	**66.70**	**73.90**	**75.91**

**Table 4:** Performance of COMTOP scheme in CASP13. Top-L/2 and top-L/5 overall accuracy is shown for 30 FM/TBM and FM domains. Targets are ordered by domain classification.

Domain	Length of Domain	Classification of Domain	Accuracy of Top-L/2 (%)	Accuracy of Top-L/5 (%)
T0950-D1	342	FM	63.2	75
T0953s1-D1	67	FM	92.9	100
T0953s2-D2	127	FM	68.8	33.3
T0953s2-D3	77	FM	28.6	0
T0957s1-D1	108	FM	61.1	50
T0957s2-D1	155	FM	70	70
T0960-D2	84	FM	63.7	60
T0963-D2	82	FM	80	100
T0968s1-D1	118	FM	96.8	100
T0968s2-D1	115	FM	100	100
T0951-D1	266	TBM-easy	97.8	100
T0960-D5	105	TBM-easy	100	100
T0963-D5	94	TBM-easy	100	100
T1003-D1	434	TBM-easy	88	89.1
T1016-D1	202	TBM-easy	97	100
T0954-D1	336	TBM-hard	100	100
T0957s1-D2	54	TBM-hard	33.3	50
T0960-D3	89	TBM-hard	100	100
T0963-D3	93	TBM-hard	100	100
T0966-D1	492	TBM-hard	84.6	91.9
T1009-D1	718	TBM-hard	92.7	100
T1011-D1	280	TBM-hard	95.8	100
T0953s2-D1	44	FM/TBM	75	100
T0958-D1	77	FM/TBM	84.6	100
T1005-D1	326	FM/TBM	89.7	91.3
T0960-D1	32	not evaluated	0	0
T0960-D4	64	not evaluated	38.5	33.3
T0963-D1	31	not evaluated	0	0
T0963-D4	64	not evaluated	30	33.3
T1011-D2	160	not evaluated	84.4	100

**Table 5:** Comparison of COMTOP model performance with a few state-of-the-art systems for 20 domains.

Methods	Top-L/2	Top-L/5
RapterX	85.92	93.37
**COMTOP**	**84.02**	**88.87**
Yang_server	77.16	92.24
TripletRes	76.98	88.61
ResTriplet	75.47	87.63
DNCON3	52.24	64.87

**Table 6:** The prediction accuracy of COMTOP with seven individual methods on CASP14 target domains.

Methods	Top-5L	Top-3L	Top-2L	Top-L	Top-L/2	Top-L/5
PSICOV	08.58	09.23	09.61	11.76	12.88	14.46
plmDCA	08.06	08.35	09.00	11.78	14.38	19.95
EVfold	09.90	10.90	11.48	10.86	13.49	17.36
CCMpred	09.02	10.29	11.89	14.97	18.92	24.34
NNcon	17.61	18.88	19.73	27.54	34.47	43.99
PconsC4	22.32	24.76	30.87	41.50	65.62	69.46
DeepCov	22.00	32.19	38.24	48.64	67.65	71.86
**COMTOP**	**41.27**	**46.92**	**53.10**	**61.18**	**68.33**	**77.49**

**Table 7:** Comparison of COMTOP’s performance with a few state-of-the-art methods on the CASP14 dataset.

Methods	Top-L/2	Top-L/5
TripletRes	76.07	83.45
DeepPotential	73.31	81.23
**COMTOP**	**68.33**	**77.49**
tFold	67.56	77.11
MULTICOM-AI	66.67	74.93
RaptorX	62.34	70.12
trfold	56.74	66.04
Kiharalab_Contact	53.11	59.87

**Table 8:** The prediction accuracy of COMTOP with seven individual methods for 57 α-helical TM proteins dataset.

Methods	top-2L	top-L	top-L/2	top-L/5
PSICOV	24.75	25.21	26.83	29.17
NNcon	23.69	24.61	26.41	29.22
EVfold	27.82	29.89	32.94	37.62
PlmDCA	27.98	30.95	35.59	42.83
CCMpred	30.65	34.83	40.88	47.74
DeepCov	35.45	42.34	53.24	63.03
PconsC4	37.32	45.44	55.16	65.26
**COMTOP**	**41.56**	**53.38**	**64.34**	**73.91**

## Data Availability

Not applicable.

## Supplementary Materials

The following are available online at https://www.mdpi.com/article/10.3390/membranes11070503/s1, Table S1: The non-redundant list with 3133 proteins, Table S2: The training set of COMTOP, Table S3: 239 non-redundant proteins used for testing, Table S4: 30 CASP13 target domains used for testing, Table S5: 25 CASP14 target domains list used for testing, Table S6: 57 non-redundant TM proteins used for testing, Table S7: Parameters $\lambda {\left(m1\right)}_{i}$ (for the seven method COMTOP) obtained from the overall training process are shown in columns 1 to 6, respectively.

## Abbreviations

MILP	Mixed integer linear programming
CASP	Critical assessment of protein structure prediction
PPV	Positive predictive value
L	Length of the protein sequence
PDB	Protein Data Bank
TP	True positive
FP	False positive
MSA	Multiple sequence alignment
TM	Transmembrane protein
