# Supplementary material for: COMTOP: Protein Residue–Residue Contact Prediction through Mixed Integer Linear Optimization
Source: Membranes (Basel). 2021 Jun 30;11(7):503. doi: 10.3390/membranes11070503 (PMC8305966; doi:10.3390/membranes11070503)
Supplement: Supplementary file 1 [file membranes-11-00503-s001.zip › membranes-1215831-SI.pdf]

membranes

Supplementary Materials

COMTOP: Protein Residue–Residue Contact Prediction through Mixed Integer Linear Optimization

Md. Selim Reza [1,2,†], Huiling Zhang [1,2,†], Md. Tofazzal Hossain [1,2], Langxi Jin [3], Shengzhong Feng [2] and Yanjie Wei [1,2,*]

1 School of Computer Science and Technology, University of Chinese Academy of Sciences, Beijing 100049, China; selim@siat.ac.cn (M.S.R.); hl.zhang@siat.ac.cn (H.Z.); tofazzal@siat.ac.cn (M.T.H.)
2 Centre for High Performance Computing, Joint Engineering Research Center for Health Big Data Intelligent Analysis Technology, Shenzhen Institutes of Advanced Technology, Chinese Academy of Sciences, Shenzhen 518055, China; sz.feng@siat.ac.cn (S. F.)
3 Department of Computer Science and Technology, School of Computer Science and Technology, Harbin University of Science and Technology, 52 Xuefu Road, Nangang District, Harbin 150080, China; 1904010508@stu.hrbust.edu.cn (L. J)
* Correspondence: yj.wei@siat.ac.cn
† These authors contribute equally to this work.

Citation: Reza, S.; Zhang, H.; Hossain, T.; Jin, L.; Feng, S.; Wei, Y. COMTOP: Protein Residue–Residue Contact Prediction through Mixed Integer Linear Optimization. **2021**, *11*, 503. https://doi.org/10.3390/membranes11070503

Academic Editor: Marco Lolicato

Received: 25 April 2021
Accepted: 25 June 2021
Published: 30 June 2021

Publisher's Note: MDPI stays neutral with regard to jurisdictional claims in published maps and institutional affiliations.

Copyright: © 2021 by the authors. Licensee MDPI, Basel, Switzerland. This article is an open access article distributed under the terms and conditions of the Creative Commons Attribution (CC BY) license (http://creativecommons.org/licenses/by/4.0/).

Table S1. The non-redundant list with 3133 proteins.

7ODCA	3BS4A	4IN0A	2R8EF	5E9NA	4DYQB	5NAKB	3B4QB	5Y7FA
4XBAB	1PZ4A	1I4UB	3GE3E	2E5FA	3OHEB	4AVSE	2ORWA	4Q4W1
2VK2A	4YWAD	4IIKA	4HU2A	3U7QC	3N0RA	4GA2A	4ABLA	4V1SB
4C5CB	1YG9A	3KZ5E	5W8QB	3OGNA	4LDVA	2W6AA	4FCHB	4WWFA
5LUND	2UZCD	2XTPA	5LF9A	1TZPB	2BFDA	3CKKA	5A0DD	1YD0A
3KYZA	3COVA	4DK2A	3QHPB	4C68C	2QMLA	4U9OA	5VHGA	4NYHC
5LU5D	5JBXA	4RUQB	1KNGA	4HE6A	1OAIA	5O1LB	2PKFA	3PE6A
4G7XA	2CARB	4XQCA	4IX3B	3WURA	3ULTA	4YORA	1VYIA	4J5RB
1WVFA	3ZVSC	2O0AA	2P51A	1WPUB	3LAAA	4KT3B	1DP7P	3FJUB
3ZSJA	3DO8A	3NO2A	3IP8A	3AJDA	1NU0A	2CCQA	4U9HL	4HI7B
2GUVC	6B9HA	5J1JA	2VB1A	3R41A	3HWUA	4TKCA	1WB4B	4J6OA
3A5FA	3QPAA	2UU8A	5AIGB	2HP0A	5MJRA	3S83A	4OUSA	5CVWA
3L1NA	5L0VA	1XDNA	6ANZA	1I2TA	3E0XB	4WY4D	4L57A	2X5YA
1OD3A	1DG6A	4R03A	4UYRA	1TP6A	4I8HA	1X54A	5HTLA	2CXNB
5FTBA	2GKGA	3U9RB	4OQVA	3AJ7A	5FBFA	2GZSA	1GV9A	5VGLA
3M0ZA	3DLCA	3GZBF	5IWHA	5L87A	1UGIF	4G1QB	5FISA	4HJIB
4UQZB	4H4NA	3KKFA	5CDKA	4CHIA	5D4VB	3DB7A	2HX0A	4TMVA
2GPIA	4FN7C	2BOGX	1BX7A	3CIJB	1KT6A	5KO5A	2ZADD	3AYJB
4U5HE	4YTWC	4QOSA	4DN7A	4RLZB	3PVHA	3TBNA	5GQIA	4B21A
4ZLDA	3RO3A	4RPMA	5II8A	4Z0YB	2CKKA	3IP0A	5D7UB	3QNSA
2A35B	4B5OA	5IXHB	2XOLB	3GOHA	3FYNA	3EKIA	5LXEB	3MBRX
5B1AK	2FRGP	4V4MT	1FCQA	2PQ8A	4GWBA	5I2HB	3D32B	2PYXB
2QE8B	4L8PA	2ZNRA	5NRMA	5HUBA	2GUIA	4LPQA	3H8GC	6C4QA
5NZXA	3D59A	3X0TA	1R6JA	3KFFA	2P5KA	3C8CB	3JQ0A	2WURA
5I7SA	3MR0A	2DWUC	2C3VA	5IX8A	3A0YB	4P0TB	1GNLB	4G4GA
2OLRA	1UWKB	4RAYA	5FA8A	3NSOA	3LD7A	6CR0A	2Z51A	1XLQA
2BZ1A	5GGNA	3S9XA	4UQXA	3GKRA	3B6EA	2QR6A	5N6FA	4RYOA
4N67A	4HDDA	2FCWA	3LHQB	6B9XB	1C4QD	4ZY9B	4GZCA	3M73A
3UB6A	1VK1A	3URRA	1TT8A	2FMAA	3S44A	1GKMA	4KT3A	3LAGA
3SMVA	3ZUCA	5AE0A	4YTKA	3KPEA	2IBDB	4BK7A	5AGIA	2P0NA
3PHXA	4UFQB	1M4LA	4GMUA	5A8JA	4XMRB	4B1MB	2DJIA	5LNDB
4WRIA	3RWNB	5UUKA	1RYOA	5EP6B	4O0AA	4F1VA	4WWHB	3CP7B
1MJ5A	6CB4A	2Y78A	3PMSA	2RFRA	4R6HA	3SO6A	4LUPC	3GNZP
3ZNVA	1T61E	5FQEB	4HFSB	3VENA	2NRRA	2ODIB	4K12A	4U98A
3WA2X	2O2XA	2IUWA	2FJ8A	1OK0A	2ASKB	3PIWA	4RXVA	4AY0A
2BZVA	3F14A	4QI3B	1WWIA	4M1XD	4EUOA	5C30A	1MWQB	2FVVA
5EHIC	2RFVA	3IB5A	1ZCEA	2V4XA	4UTUB	2YN0A	5NGGA	5GNFB
3NO7B	3D7JB	4Z80B	5J1NA	2X49A	5EL9A	5NJ9C	2I5UA	3DK9A
5VXVA	5EWOA	5I95A	1QV1A	4GT9A	1W4SA	3NKEC	2GUDB	5HEEA
5LEOA	4BOUA	1BKRA	1SZHB	2R16A	5KVSB	5J3TB	3A9SA	1ID0A
4KV7A	4CS4A	3SZ3A	3TT9A	4Z0GA	5DT6A	1MN8C	2NNUA	3SGGA
5YJ6A	1XQOA	1K7CA	1TUAA	2YKZA	3AJ4A	4CDPA	5UDIA	3T7HB
2I53A	5UAMB	4ZCEB	2OVGA	5WUCA	3L9AX	5BTYA	4YI8A	3L8WA
4YUCA	4QPNA	1ZK5A	4M0WA	3RNQA	5TC6A	2JKUA	5F4CB	3ISXA
5UEJA	4ZJHA	3VJ9A	4P5NB	5M29B	4ZILB	5O5SA	4PXYA	3V5CC
4UE0B	2I49A	4I4OB	5XDHC	5DZEA	3ZN4A	1VMHA	3T3LA	1SVFA
2F46B	1WERA	3R2QA	4WN5A	5OL4B	2ENDA	1RTTA	2NLVA	1V4PC
1ZHVA	5W2IA	3R8JB	3ZBOB	2HIYA	3MDQA	4JMPA	2O1QB	3DKRA
3IE7A	5F7FB	1VKKA	4OFAA	5LPAA	3ITQB	5FVND	4G9SB	3ZSUA
4G6TA	6BLMA	4XDUA	5B5LA	2TPSA	5XP6A	2CC6A	3BQPB	2R5OA

5B1AD	5OVOA	5A1IA	5VBDA	4L9PB	1NNFA	3ZY7B	4ZV5B	3M66A
3HNYM	5C9OA	5TPIA	3MC3A	3EQXB	1WVQB	2IZRA	1A62A	4P7XA
3H75A	3BEXA	1W0HA	4M9VF	4XXXA	6FG8A	3OBLA	5E4BB	4WKAA
5FYPB	4V3IA	1FSGC	4YZ0A	1HZ4A	1UUYA	3LLOA	3QZBA	1ZZKA
2GJ3B	5CTMA	3ZW5B	2QL8A	2XZ2A	4XQ7A	5KF9A	4PZ0A	2FHPA
5IHFB	2V76C	4OE9A	2G7SA	4I6RA	3RFEB	4R6YA	2WF7A	5TW9F
3SQZA	1U8VC	2VCLA	3BUXD	2BT9C	4DRIB	4RCJA	2NQWA	3NOHA
1FO8A	3OE3A	1RV9A	1O8XA	1ZL0A	2CXAA	3SSXN	3JUMA	4WPYA
1IX9B	1IRQB	4RD7A	3MVSA	3W06A	5I34B	3G46A	3ZZOA	5U69A
2DKOB	2COVD	2WDCA	1Z6MA	4AYOA	3A8GB	3H7IA	4CP6A	3S2RB
4N13A	5L37C	3OV9A	2Z6OA	5NW3A	5KDSA	5O8MD	3EDHA	4KQPA
5GJIA	2INWB	4GT8A	5AZBA	5I5NB	4BT7A	4AF8A	2POFA	1JY2R
3FSSA	1LNIB	5V3NA	4GC3A	5UQ6A	3CECA	1GCIA	4B0ZA	2FSRA
3I94A	2PHNB	4MNCA	3FG9C	5VEOA	1KMTB	4PI8A	3BLNA	5J41B
5XA5A	3F43A	1EUVA	2QLTA	3O8MA	3U6GB	5JVIE	3MYXA	3OCJA
3EF8A	2X5XA	2ZA4D	4ACJA	1MC2A	4EICA	5UGRA	3GNLB	2PC1A
2WLVA	4LRUA	1V7WA	5X9IB	2W91A	3MQDA	2F9HA	4YECB	4R75A
4DM5D	4WZXA	3IEZA	4NTDA	2VOKA	5E9PA	5B1AJ	1NC5A	5T3BA
3VL1B	2ZHJA	3DJEB	5LBDB	1G3PA	4V1GA	1EZGB	4HBZA	5UBAA
5J4OA	4J42B	6COJA	4UQWB	4U89A	2O9UX	6EQEA	4WHSC	4QBOA
5DM2A	5FKTA	4NKPD	4EE6A	2R6JB	2Q5CA	4ZQXA	1T3YA	3PB6X
5A6WB	3PPLA	4BYZA	4A2VA	3WKGA	5MY7A	5B8DA	5H0MA	2BU3A
4KRUA	2CIBA	4B9GB	5UXMA	4OQ9N	4PSRB	2X9ZA	6B9XC	2CG7A
5KVRA	1Q6ZA	1DK8A	6CD7A	5COZA	5M7DA	1JG1A	4J7QA	3CL6B
4CNNA	5OLRC	4G8BA	1E58A	3ZRXB	5MX9A	3Q6BA	3LFKB	5BMNA
2WTPA	4LRTC	2GLZB	1GMUA	2OLNA	1MKKB	5IPYB	5A6WC	3O12A
2C92E	2FHZA	1XG0D	1GPPA	5UX1C	3O7BA	5BR4B	3A57A	4MCOB
5LY8A	2IA7A	3GKJA	1F9VA	1BGFA	1NG6A	1UI0A	5BOWA	3RRIB
5HB7A	3QVPA	2NR7A	1EAQB	1Q0RA	4UU3B	2D68B	1NYCA	1LS1A
5LUSF	1Z0WA	4O6UA	4Q4GX	1L9LA	1FYEA	1M2DA	4M7TA	2JISB
5WK0A	5ICUA	1GY7B	4XOTA	5A71A	2JAEB	5M33B	4REIA	3RQTA
4Y9WA	5V1YB	2QEDA	3VQJA	3W7TA	3K6YA	4P40A	5DICA	4N1IA
4F01A	3IISM	3VLAA	3M7AB	3CT5A	4PWWA	1GP0A	2VFRA	4I66A
4A3ZA	4JF8A	5HTXA	3KEVA	3L46A	5FSVA	1EUWA	6B1KA	5B08A
1M15A	2O7AA	4PXEA	1K5CA	3TG0D	5SV5A	4GNRA	1KAFC	4MYDC
4P3HA	1C1DA	2P8IB	4IILA	2D5MA	4Q53A	3BMZB	5T39A	3X2MA
1CCWD	1DJ0A	4EBGA	3O2RA	3JU4A	3K5JA	1JKEB	3SBMA	3A02A
2HHCA	1DS1A	3GO5A	2OB5A	1W1HC	4ZVCA	6BHDA	4A29A	5JAWH
6C3CA	5KO9A	1MUWA	4AW7A	3GOEA	1VE4A	5EDFA	3X34A	4HFQB
2QGUA	2EGVA	4FR9A	3BWHA	5CR4B	2EHZA	4A37A	4YE7A	5XDCD
4MNOA	2XN6A	5OPZA	4HS2A	3MCWB	2CJTD	5W83A	2OOAB	2RKQA
5JJ2A	3LO8A	4Z47A	5JXMA	4YSIA	3EUNA	2VXNA	1NC7D	3HDXA
2JHFB	2NZLA	1MNNA	1VYKA	3U65B	4LWUA	2BAYF	2C61B	4INWA
4ZOYA	2I51B	3LYHB	3S6EB	2QNGA	5NAIA	2CE2X	3H4OA	4BN4A
5JH8A	4KEFA	3NJNC	4TKBA	3P4HA	1OCYA	5AGRA	3U5SA	5C5ZB
2GB4B	4QXLA	3R4ZA	5C40B	1RG8B	3UJCA	5B5ZA	3IVVA	5G3YA
2HLYA	2OA2A	5CKLA	4V33A	5EPWA	5M0NA	5WS7A	5GMDA	2FWHA
4CGSB	1QNRA	3S4EA	2BHUA	5LQ6A	3D9NA	2B9DB	2QXFA	3BF7A
5BY5A	4CD5A	3FTDA	4WJQC	4WJIA	1WTJB	5I45A	4X5PA	5SV2A
4BPSA	4G10A	4M51A	3DGTA	5B1AI	3MVCA	2RKLC	1CXQA	5J3TA
5COFA	1KQFC	1U07B	5BS1A	1R7JA	2YDTA	5DP2A	1GK9A	5K4XA

5K87A	3NR5A	4NESA	5OMTA	3D3BJ	4OCVA	4L9PA	3GY9A	2YIMB
5A61A	5DCUB	3BT5A	1ZGKA	4FUUA	1TBFA	1XBIA	5VGBA	4MTMA
2HQSA	5CGQA	5AOTA	2XHFB	5I39A	1IUQA	1V05A	5YQJA	3W0KB
3ZJAA	3MILA	3H4TA	2W1JB	3GWIA	2PYQD	5KTNA	5K8JC	5DU9A
3M0MD	2WFWA	4EZGA	4ONRA	1J77A	1OQJA	3KYJA	3LDCA	2XIOA
4OH7A	4LTTA	3G5TA	2QNLA	4Q4W3	4P7OA	1VD6A	5VGBB	5M72A
4FNVA	3G21A	1OW4B	4MUQA	2W1SA	4UNUA	5COWA	3A8GA	3UIDA
2BLNB	5KVGE	5F2KA	5JEDA	3B64A	3ZHOA	5FFXC	3BY8A	2HUHA
4RD4A	1SQSB	4ZBHA	3WIWA	5CGQB	2QNKA	4D8BA	3CXNB	2IAYA
2PRVB	3MJOB	3RT2A	1WCWA	3KM5A	4NMWA	2IMHB	4J8SA	2FVYA
5NSAA	3VIIA	4W7LB	3EOJA	2FI1A	3I4GA	3DSKA	2DSKA	3CBZA
2FCWB	3I2KA	4MIYA	3MXNA	1E7LA	3T7LA	5YDEA	4DQJB	3SUUA
4DVCA	4I1KB	5B1AE	4WSFA	1H97B	4A4JA	5TVOB	5IDHA	1U7IA
5MYNA	2WK1A	3WI9A	3PD7A	2BBRA	3M6ZB	4Y1WA	4PZ3A	1Z70X
4P3VA	4EBJB	5G5CA	2W3QA	5HHED	5JELA	4AFFA	4D0UB	3DQYA
5EJXA	4IX7B	1M1FA	2RE2A	3NBMA	4BQNA	3ONDB	2Y9UA	5EWYA
5WD9A	2F22B	2A0BA	4LF0A	3Q4OA	4YDRB	3WX7A	4J0DA	4DOIB
4YG0A	2WZBA	2OCTB	3R87A	5WLFA	1DD9A	2CI1A	5AMBA	5PMXA
4YYCA	5MY5A	3EYEA	1RWHA	2IC6A	4JB3A	4BU0A	2QZCA	1YQSA
4ME2A	3N2WC	3IMKA	2DKJA	4V1JA	5IXBA	1XSZA	2AIBA	5NHUJ
1TKEA	3MSTA	2J73B	2F23A	3OCUA	4DQ9B	1J0PA	5O63B	5XJ5A
3BONA	5B7HA	3MZFA	1DFMA	2VLAA	2G7OA	6FDGB	1WMHA	5MAWE
4CJ0A	4PH2B	4C6AA	3MCXA	2G3RA	4L2HA	5CWHA	3A35A	5K91A
2O9SA	2HMJA	3Q46A	4FK9A	2J43A	4WZ4A	4WPGA	5AOZA	2OIZA
4ES8B	1OF8B	2RBKA	2J6VB	4PJ2B	1I1WA	4P3AB	6B2VA	2AKZA
4ZX2A	2DPLB	2XW6C	4PVKA	4S39A	2X5NA	4XFKA	3O2TA	1US0A
3Q4AB	4EGUA	2Z26B	3SK7A	5MDUA	4EAEB	1JO0B	3GDMA	5ZHOA
1FP2A	3VUPB	2Y24A	4C08A	5EL3A	3BM7A	3GMXB	5YALA	3VSVB
3HR6A	5LZKB	4WY4A	2UV4A	5O58A	2Y6XA	6ES9A	3SZYA	5D1MB
5HWAA	5JBNB	5E75A	1N3LA	5FLWA	3S5MA	5JDKA	2Y0OA	3A2ZA
5A3AA	4L2IA	4E15B	4QXBB	5O75A	4G3OA	4NYQA	4LLDB	2CYJA
3RKGA	4DT5A	1W53A	3DXLA	2OXGD	3MJFA	1R29A	1RA0A	1JUHD
4JJAA	5CD2A	2OFCB	5M7YA	2JLIA	1LC5A	1S1DA	3FRHA	4DMID
1OYGA	1VL7A	1H2CA	5AEZA	1C0PA	5HZDA	4KH8A	3U7QB	1VKEE
2EABA	1XG0A	1GWMA	4AQOA	2WWEA	2V25A	5WPSA	1W5QB	5M2OB
2V89B	4Q2SA	5M0WA	1UWCB	3FXAD	3CCDB	1ISUA	2X4LA	1T8KA
5N41A	3ZZPA	4HMSB	4HSTB	4WQKA	5A1QB	3NVWB	1S3CA	3SJMB
3QZXA	4PIOA	1TQGA	3ZDBA	3LM3A	4QHWA	5XK6B	4YAAA	3IUOB
5O0SA	2YH6C	4PSFB	3GIWA	5LP9A	4EQPA	2V8FA	2V33B	4DZIA
4GXWA	3NYCA	3R9FB	4Y9VA	5MPWC	4NDSB	3NVSA	3WJTA	1ODMA
4NPDA	4HTUB	1VQSB	5H3VA	4IFAA	2RILA	4CICB	3ZN6A	2IMFA
1ELKB	5H7EA	3SU6A	2PNEA	1T9IB	2FCJA	4IYAA	3BVXA	4H7WA
5LJMA	2J5YA	1W7CA	3RPZA	2JEKA	5V2OF	4W7WA	4U7AA	2QSWA
4PLZA	3LHCA	4BFOA	3C70A	2IMQX	2GOMA	1Z3EB	4E29A	4X2RA
4Q2LA	1RYLB	3DHAA	4M9KA	1HXIA	3ZBDB	3BGUA	2FUPA	3E8TA
1N13E	3H6JA	3KU3B	3GE3A	3W42A	3HLZA	3S8SA	4K7BA	3CJSB
4V1KA	4KL0A	5X5MA	4R78A	3SNOA	4U8FB	3HS3B	4PJ2D	3VGIA
3Q1XA	3H0NA	4CJ0B	2QB7B	2EHPB	3F9XD	3UFFB	5TIFA	2EW0A
2Q3TA	2ZCMA	2H8EA	1FM0E	2V9VA	5N0OA	3ZZYA	4BGBA	3VWNX
4D7CB	3N6ZA	4X1ZB	3V68A	6EIOA	3PFBA	4ZZ1A	4YPOB	3HP7A
1LUCA	6B9XA	1JF4A	4R5RB	3PN3B	1F86A	5NCJB	3FILA	2OKTA

4O1RA	5AIMA	5D8VA	5TJZA	4Y88A	1VHNA	4Y0GA	2YH5A	4IAUA
4ZOTA	3VZ9B	4I84B	5K8JA	4A56A	2OV0A	2P2SB	3H7CX	1ZI8A
1WN2A	3CUZA	4ZBGA	3NFTA	5ZBYA	1QG8A	1LZLA	1XMKA	4UA6A
3ZR8X	3H87C	2XJ4A	3PFGA	4H6QC	1F1EA	5L9ZB	5W8OB	4B6GA
4U0OB	5MFOD	3MMHB	4W8PA	1O7JC	5BY8B	5LS4A	4MF5A	3SC7X
4EYZB	1C7KA	2JE6A	3P02A	2F62A	2XEPB	1S9RA	4G0XA	5TZMA
1K5NA	2FCTB	5B1AP	5DMAA	2ERFA	2GUHB	3ZXCB	4HLYA	4Z9HA
5Y2SA	6EOZA	3LB2B	5DUTA	1VJUB	4YFUA	2W1RA	1G6XA	2ZUXB
1H99A	4XPXA	4HWMA	4XXFA	2CZSB	4PQDA	4QXBC	1NUYA	3L81A
3GOCB	1IFRA	4YZZA	2W5QA	1LMIA	2I3DB	2OFKB	1KQFA	2IUMC
4MYKA	4I71A	3WS7A	5K8SA	2QSBA	3ME7A	2IXMA	4LX2A	4UDXX
5JI7A	3WGXA	4F2LA	5V44A	1D5TA	5TRQA	1GMXA	3WMVB	5JUHA
5J0KB	5JS4B	5UOUA	5BMTA	3WCQA	1EGWC	3B9TB	2W7AB	3LYDB
5UE1B	5CL8A	3B9WA	4ZHWA	1NXMA	1W5RB	5VCMA	2X5OA	1HQ1A
3POWA	3S6FA	1X6IB	2R9FA	4Q98A	3H74A	1NWZA	4YNXA	3D06A
4H4DB	1T5BB	2AXWA	3MXNB	4W8BA	4Q4W2	4MFIA	1Z67A	2IBNB
5F6RB	1C5EB	4UE8A	5LB7B	5M12A	3TG2A	2RDQA	4XJ5A	2C2UA
2PQ7A	5C2UA	1S9UA	4WY4C	3PL8A	1QV9A	4PF3A	4LGJA	3JSYB
5J1SA	3MDUA	5GNGB	6C2ZA	5X7LB	4X2UA	3D1PA	4YNHB	5CEGC
3K05B	4JZZA	3ZZSG	2NSZA	3POHA	3D3BA	4V0KB	4QTCA	6EHIB
5X57A	1RKUB	5JAZB	4CA1B	5D66A	3OYVA	5W2FA	3PIUA	3IX3A
4NBPA	3X0IA	3W0OA	2A26A	3UR8B	5NT7D	4GMQA	3F1LB	4RVQA
4H5IA	2YV9B	4E4RA	2RBDB	4XEMA	5KFZA	4EP4B	2VWSA	4QKDA
4BQYA	1MY7B	2ANXB	4GS3A	4BJ0A	1H80B	2E3HA	3TYSA	3WQCA
4JM1A	4WH9A	3FGHA	1RP0A	5V6JC	3BB0A	4E6FA	5CECA	1YFQA
4W9ZA	4NI6A	3JS8A	2W31A	2CIUA	5O37A	2NMLA	3RPEA	5A62A
4JDUA	4HLSB	1WPAA	1F2TB	1J3WB	4TSDB	5X4RA	3TU8A	3WQBB
3A16C	2HW2A	2IP6A	3Q2IA	1QS1A	5XVEA	5HHJA	5L77A	5DWAB
1OU8B	5WSFA	3IPJA	3UFEA	5ELBC	1RTQA	3BI1A	4AL0A	3TM8A
2YMVA	4X9XA	3C9UA	5OF1B	4K12B	5E5YC	4I3GB	4DJAA	2QJWD
3MABB	5HNVA	5C17A	4WFOA	5KLEA	5VYQA	3ROFA	1XD3A	2VE8F
2APJD	1F2TA	2NUHA	3KC2A	1W0NA	2Q2FA	5OL4C	2ASBA	4BVXB
5THXA	2NVHA	3KIZA	2QJLA	2VQ2A	4Y9IA	3IKWA	4JBDA	4RRIA
4XZFA	4JEJA	1M22A	1KWFA	3IQTA	5LXXB	3FKEB	3TJMA	2WNKA
2WE5B	2J9WA	4HC9A	3ZHIA	3PLWA	5VNYA	1E5KA	2W3GB	4EMTB
1HDOA	3SIGA	2I5VO	1KKOB	4OJXA	4EDHB	1WT6D	3WH1A	5GV8A
4LD1A	1K3XA	2IT2A	5JO8A	4RFUC	5V01A	2X2SA	2BDRB	4CC2A
5A0NA	1ATGA	3SZVA	4XINA	3BO6A	3L6BA	4QM6B	2CDPA	4JHTA
4MXTA	4BVXA	3VMKA	3CMBD	2ZQ0A	4MVKA	2XHGA	6EVGA	4WY9A
5N8AX	6FFAA	2ZPMA	1XUBA	1V5VB	2HEWF	4UPIA	1VCCA	3S8MA
6CD9A	5W98A	3K67B	2PRXB	4F06A	3QR7B	5U3AA	4JCCA	4WIQA
3G7RB	6BJBA	3Q1CA	2GZ4C	2FP1A	2OQZA	5DHDA	5TT5A	3T4LB
4JGIA	2OFZA	4F54A	3GPIA	4IC4A	5M1MA	5HQHA	1G61A	1WZDB
4A9VA	4TZ1A	4CVRA	4E40A	5MAOA	2GKEA	4F87C	4HI8A	4PH8A
4LJOA	3G02B	4H14A	2R4IB	3RPCB	4YWKA	5H1NB	4N02A	2XQQC
5JGKB	2W7ZB	3U3LC	3JRVA	3BHWA	2E7ZA	2XPWA	1Z72B	1N62A
2BWRA	5OL4A	2OVJA	2XBGA	1VBWA	4JP6A	2DDXA	2DTJA	5DLED
1O7IA	3C9AA	1QLWA	1YD9D	3T92A	4S28A	5G38A	2GGCA	1KYFA
2VZCA	4R81A	6EUWA	3AGNA	3M9QA	1K7JA	3K7PA	3KWRA	3C2UC
1JX6A	5EW0A	4WPKA	1NKIA	4Z3GA	1N8VB	5JUGA	5AQ0A	1E6UA
2RL8B	2OMLA	3IWFB	1M9ZA	3B5MC	4BRCB	4NN2B	1R5MA	2GS5A

4ZV0A	3FSOA	1LQTB	4HWVB	3EURA	4WNDB	5K1PA	3L51B	4CO8A
4XFJA	3AMRA	1USMA	1H32A	2HBAB	4PQQA	1JM1A	5O2XA	5K2XA
2X46A	3BUUA	2A3NA	2Q6KA	5O15A	4KQIA	1HFET	3I10A	5DVIA
3MEAA	4Q4W4	2VEZA	1G2RA	2B0AA	1Y8AA	1FCYA	4XD1A	5XN3A
6AMGB	3EJFA	5A0YA	1YPYA	2CIOB	3ZYPA	5DGJA	4E6UA	1SH8A
2ODKB	5TDAA	3UXJB	4WBJB	4RUWA	2FULB	4KU0B	1Y5HA	5CPHB
4XUWB	4ZMKA	2QUDB	1OI0D	3M3PA	3CKCA	3S2JA	2O90A	2YC3A
2QRLA	5CEGB	2ILKA	5H28A	4DI9A	1GUTE	4NSVA	2HEUB	3PLNA
4YECA	4X84B	2WH6A	1C1KA	5VPUA	4RJZA	1LC0A	2DEJA	3V0DB
4XFMA	2WNPF	5ML3B	4YCBB	5U81A	2H30A	1RXIA	5W0GA	3WWCA
5TKWA	1NZJA	5OE3C	5C5GA	2V8TB	1X91A	3H3LB	4YX1A	5LT5B
4PDYA	4PSSA	5MGWA	1IQZA	1JNRA	3FN5A	1QWGA	5B1AN	5AZXB
3HYNA	2JG0A	2XODA	4TTWA	3G36C	4YQDA	4ZGFA	2ZWSA	5J1SB
4F0WA	3D9XB	2Q9KA	3PESB	3HPCX	2ABSA	1F8EA	1IOMA	5HL3A
2RHWA	2Y8YA	1WDDE	5EZUB	2JE6I	2CZLA	4M91A	4EA9A	2R31A
4KU0D	3BOEA	5XEVA	1HZTA	2P4FA	5UWZA	5HJ9A	1P5ZB	4E3YB
3CTZA	5LW3A	3N10A	4NF1G	2P6WA	3DS4B	3POJB	5A95B	3N01A
4OD6A	1I27A	3RX9A	4KX4A	4NZKA	4OA3A	5T1IB	3MQZA	4XEZA
3D4EA	1LQVA	2YVTA	2VLQA	2O0MA	2WDSA	5DMDB	1YS1X	4E2XA
1X9IB	4R9PA	2BFDB	4I6YA	3KE7B	3TUTA	5K3XA	2V8IA	5X9LA
3TG7A	5JRYA	1UNQA	4XDXA	4W78C	6FNUA	3D0JA	6AVXA	3LSNA
4C84B	3EERA	1J98A	4D0QA	1JB3A	4RJWA	4MBYF	4PNOA	4X9RA
4EVUB	3BWZA	3KUVB	1Z3XA	3PUCA	1WPNB	5VX1B	3A6RB	4CBUG
5FPZA	2ZPTX	3MJEA	4H6CI	5FAFA	4ZAVA	5TSQA	4UHTB	4RP3A
2IYVA	2VNGA	4YMYA	1INLD	1J3AA	4MZJA	5DJHA	5BPKC	1SAUA
6F8PA	1PMHX	1DCSA	4EQBB	5NZGA	3QP4A	3D40A	4DWRA	1EVLD
5EMIA	5LJXA	3I33A	5BJXA	2NN5A	1EJ8A	4JTMA	2Q1SA	5G51A
1YNPB	5D2KA	4UHQA	4DO4A	5IDVA	3GAEA	2CDCB	1N13L	2YLNA
3IV4A	1JZ8C	5EMXB	5OHQA	4ZR8B	3B0GA	4FBJA	2WQFA	3SD2A
5MBXA	5J93A	3FGVA	3F0DF	3QHBB	3ELFA	3TDUD	1QW2A	2HX5A
4GHNA	1V5IB	4V0SB	1GVPA	4AT0A	3DXYA	5NWPB	1JHJA	5YGBA
4X9ZA	2WOYA	1NTHA	5APGB	1R45A	5ERQA	4RDBA	5EQ7A	5SY4B
1RK6A	3TEWA	3MAOA	4EQSB	3D2QD	4G54A	4S1PA	5KARA	5TLEA
4PS6A	6F0QB	2CIWA	6B6UA	5OL9A	4DMVA	5KVBA	5XBIB	2PR7B
6B5KA	5FMUC	5BPKA	4W78H	3EA6A	4UOBA	1VR7B	5LHMA	5ECKD
4BOQA	3ZOQB	5IG6A	4LGYA	3LFRB	2UVKA	5O9MA	4WCKA	3BEDA
3LHNA	4GWGA	2P9WA	5II6A	4AK2A	1UZ3B	2VXTI	1V6PB	5IGIA
2Z0JF	2CXYA	3JYOA	2EAQA	4RTHA	1JL1A	2NWFA	1KGDA	4TR6B
4QEKA	2OZTA	4MAIA	4YGBD	2XOMA	3ACHA	2QFAB	3CHMA	3AOWC
1EKQA	4JG2A	3A09A	3VVVA	3AKSA	1Q7LB	5M0YB	4U9HS	3N08B
2V3GA	4LHSA	3T47B	4YTDA	1OZ2A	2B97A	3OBQA	3DNJA	3VORA
4A7UA	2YFOA	2BO9B	1PSRB	3TEUA	3OMYA	3WVAB	1X0TA	4M5EA
1T1UA	4CI9A	4FTFA	4YSLA	1GK9B	3GZRB	4WE2A	5FYDB	4ZH5B
5MFAA	2VC8A	2FR5C	4L2IB	2DE3A	4CD8A	5F5NA	3DKMA	2J5GD
3B5NC	3CJMA	2NWRB	3HKWC	4N2KA	1GWEA	2DY0A	4N2PD	5I90A
3RY4A	2FYFB	1M0KA	6CBNA	5XVTA	3D1KA	4EFPB	1XPPA	5IHWA
2E4TA	2F60K	2ZHPA	3R6DA	3HJ4B	3G0KA	5MPRA	1RFYB	1ZV1B
3TOSJ	5DFSA	5AGDB	4BJIA	2BRYA	4L0NB	5OD4A	2PXXA	3LLUA
2WOLA	1Q5YC	5J80A	4CV7A	5XM5B	1SZ7A	5IO9B	3U2UA	1N08B
4QY7A	1PO5A	1XMTA	4IPUA	3PLUA	7A3HA	4PE3A	1RKIA	3H79A
4W8HA	4QPTA	4M8AA	3BHQB	2OPCA	4Q7OA	5FS8A	6ENSA	3OG2A

2YZYA	3UI4A	3ORUA	2NXVB	4GEIA	3GBWA	4GVQA	3WDCA	2QCPX
4NOHB	5TQIB	1MK0A	1SBYB	4QLPB	2DLBB	5IDBA	2WFIA	3C8ZB
4IIYA	1GA6A	3OAJA	2XEVB	2P17A	5NVIA	6B29A	1CY5A	4JGLA
3AIAA	2OB3A	4JQFA	4LEBA	1WHZA	5KKOF	3FGYA	5B1AG	4ESWA
3OM0A	2BMOB	2WZOA	1UOYA	1U84A	2VPAA	4BGCA	5J4FA	3OV5A
5UFYA	4EKFA	2BL8C	4ZC3A	5FU5A	2CS7B	3KGKA	6FEAF	3IE4B
3OMDA	1W23A	3B5OA	2OLMA	4JK8B	3HM4B	5AHKA	2MCMA	2ZCWA
5KLAA	2GXQA	3WH2A	2H1VA	3RLGA	4B89A	3GE3C	4QLPA	4HROB
3K1UA	5AZWA	6C29B	3BC9A	2C60A	4AC1X	5JCAS	4OQPA	5J4LA
4WLHA	4IUSA	2RA9A	3WZ3A	3A9FB	5F47A	1X6OA	4ARUA	3HZPA
2UYTA	3CT6B	5OUOA	3JXOB	1JNIA	1A3CA	5A9TA	4CUAB	4BEUA
3ESSA	5GS7A	4KM6A	4R2XB	4D7JA	4O0CA	3F7EB	4FZPA	2NW8A
3B0XA	4GJZA	2OY9B	1UKFA	5UM2A	2XRYA	3NO0A	4NOGA	4LVFB
5SUIA	1V0WA	1LLFA	3FEGA	3NBCA	3JUDA	5UFNA	4B4DA	1JETA
5KXHA	3C8LB	2X9GC	4XLZB	4P5EB	4NNOA	3M5QA	4J7NA	2RAFB
3EN0A	4IQBA	3ER7A	3EJVA	3H9CA	2DXUA	2WQKB	3QC7A	4AZ6A
4WEEA	5HT2A	5P9VA	5C98A	1Z2NX	2BKXA	4X7GA	5GTQA	4PHJB
5GZ3B	3QZRA	4D0PA	5XBCA	4HHRA	1NNXA	3FWKA	1L3KA	5EQ0A
2PIEA	5A6MA	2V3IA	3QL9A	3VZ9D	1Z6NA	5IMAA	1VP8A	3WARA
1ROCA	1K4IA	4W79A	2CWSA	5JICA	4Z7EB	2V7FA	2HINB	5UEBB
1UCSA	5H0QA	2R01A	4M82A	5Y9AA	2OPLB	2II2A	4TKXL	1H4XB
3Q64A	4IEJA	4PDNA	2WY4A	1KQPB	5AMHA	3IQUA	5Y4ZA	3M8JA
3A0SA	5L74A	5GV0A	5EJ8H	4PP4A	3EOIB	1WS8B	4I8IA	3GNEB
4A3PA	3TOWA	4B1YB	3HF5B	2PVBA	4N03A	4YTBA	2R0XA	1MUNA
1EB6A	3CZXB	3E8OB	4ZRXA	4N30A	2ZOUA	1ZVAA	3TPDA	5GRQA
5JDAA	5I0YA	3SXMB	1H16A	4GVFB	2ZFDB	4HI8B	5B4ZA	4BPFA
3KH1B	2PA7B	5OBTA	2Z72A	2FBAA	4J9YB	3VUBA	4KDWA	3G91A
2HQXB	4EZIA	2AP3A	5B1AU	3HO6A	3IFEA	5WRIA	1SENA	4HVKA
2JKHL	1PP0D	2WQ4A	5A99A	1WNAA	4MYZB	5BYKA	4I0WA	5A8CA
3TKFA	4M1UA	5NOAA	5A0YE	3IT3A	1YBKB	2XVMA	3W07A	5B1AS
4FP5G	3ZOJA	4W8QA	1N62C	1V7ZA	2ZDPB	3LGBB	5H6XB	2XETB
4AXOB	5M10A	3GA4A	4GEKA	5B5IA	4M5RA	4OHJA	4ZBOD	4Z67A
4XB4A	3VN0C	2J8KA	1T6UE	4IGIA	5UMRA	1ZMAA	3L1WD	2O8QA
5NVGA	5UJCA	5XKXA	5NJ9D	5SVYA	3HZ8A	3JTZA	3I7MA	6B9XE
1CC8A	1KJQA	3R5TA	3SHGB	3LW3B	5EC6A	5BY8A	2IMJB	1VMGA
1UTGA	2FCOA	4J1OA	4ERYA	3OXPA	5NQOA	3NUFA	2XFRA	5NT7A
1R9LA	1WLZB	3PP5A	5FJDC	5A0RA	4YEPA	4TXRA	3LS9A	5B1RA
2X3MA	1IDPC	5A67A	5J6YA	1JBEA	4RZ9A	1BYIA	2VOVA	5CUOB
4IUMA	2O2KA	2C71A	3E48A	3SS7X	6AO9A	5MK9A	2HA8A	2AMLB
2D3DA	4RWUA	2DKOA	1M7GB	3CLMA	3QM9A	4OI3A	4NL9A	4R1JA
2P0SB	3LMZA	5IUCB	4BB9A	3GIUB	4NUTB	5I5PA	2EPLX	2W15A
3GP6A	5TVOA	5DKXA	2CB8A	4JXRA	1I24A	5LJPA	5C12A	1P6OB
5CVDB	2QFEA	3GVEA	1KOEA	4REOA	4RPTB	6BCBA	2BKFA	4PUXA
1KQ6A	3U9WA	2W50A	5M97A	2Z5WA	4UYBA	2V9LA	4ZV0B	2R6UA
2RK9B	5KZZA	2D1SA	5LXZB	1M55A	4WP9A	6ETLA	4QGOA	5C33B
3ON9A	3SK2B	1CCWC	3MWXA	5C6SA	3V7NA	3DFGA	2VFOA	2FKKA
4YLQT	5A0YF	4XTBA	4QRNA	2GKPA	4WUIA	3ND1B	4TYZB	3WDNA
1W66A	3RZNA	6F0PA	1UCDA	2IU5B	5Z42A	5X5VA	5YXMA	3F2ZA
1NNLB	3LFTB	2QOLA	2Z6RA	2EX2A	2GYQB	2GQTA	1WHIA	2OHWA
4NOAA	3AWUB	2RH2A	5FOTA	1AH7A	5CYVA	3GRDB	3QGUB	3SCYA
1KNMA	3WMTB	5IT3B	4QP5B	4HY4B	3FYMA	5XAVB	1US5A	3LTIA

2VH3A	4HNOA	2D5WA	3ACXA	1T92A	4GOFA	5BOBC	4DDPA	1UCRA
5K4BA	2QIPA	4TXRC	1F46B	3GE3B	5LS7D	2IA1A	4MAXC	3PF6B
3MU7A	2W47A	1LWBA	1HYOA	3U25B	3P0BA	4KZKA	5B4BB	2RHFA
1YT3A	2CNQA	2G84A	3SEEA	4AE7A	4HCJA	2QT1A	4AU1A	4RAXA
2R2ZA	3Q7RB	2WJ5A	1GYXA	2XJPA	5D5YB	5UZGB	3VMNA	5DBLA
3LT7F	5M72B	4F2EA	3KGYB	4WY4B	4LMYB	1OH0B	3AJ6B	3LHIA
3NDHB	2GRRB	5NZOB	2W40D	1NKDA	2AGKA	2NLRA	4ES1A	5U0IA
3FDEA	1KDGA	3SUJA	5O29A	4GB5A	4MC3A	1S29A	5T8CA	4COGC
1Y6XA	4FZLB	2FHZB	2J6BA	2VCHA	4WTPA	1NZ0C	1QWOA	1XDZA
2WWXB	5HGZA	2YVEB	2V1MA	4H8EA	5O6TA	5LS7B	1VLYA	3BA3A
3B79A	4NLMA	6BO0A	3LWXA	3LAXA	4C5KB	4OMBA	1QW9B	5TOQA
4AFMA	4ZPCA	3GHAA	4CFIA	2HS1B	4RY1B	5J90A	4MZCA	5A7VA
4OPCA	3VRDB	3MWZA	2DXAA	5UQZA	2FSQA	4WDCA	5HDKD	5TCBA
2FGQX	4REKA	4A02A	5ISVA	5HB6A	3F6YA	2OKFA	2YHGA	4H3UB
4ALZA	2I8TB	1K4NA	1IO0A	3GMGB	4PNEB	5JE0A	1N62E	5JEEA
4Z7XA	2XWVA	2RKVA	3O4PA	4G9SA	4JN7A	3B0FB	4TVOA	3PJPA
6COJB	1W6SC	4LUAA	4DQAA	5OQ3A	2CCVA	3BQXA	4RU3A	4LA2B
5LZNA	2ZS0C	4HTGA	2Y5PD	2BF6A	4GUCB	2PFIA	5C8ZB	3IUWB
3OFGA	3E4GA	4R3NB	5M17A	5FEWA	4EQAC	3CWRB	2YNAA	2W8TA
1Z2UA	3PFEA	1I71A	3SOJA	2E3NA	5B7YB	2IJ2A	1ZUUA	5NCGB
3QX1A	2G8SB	3TJ8B	4MJDB	4WJTA	4K82A	4OM8B	3R72A	4QA8A
1JOVA	1W6SD	4ESMA	2GJ4A	2WNFA	2AYDA	2IC2B	4JB7A	3GXHA
4QHQA	5IK4A	1X6ZA	3CI3A	1HPGA	4HS1A	5L9AA	2F01B	3P3CA
3CKMA	2AACB	1P1XB	2Y7EB	3KWEA	5JIGA	2OYOB	6ELMA	4TVVA
3FMYA	4Y2FA	2PY5A	4KNKB	4KBXA	3NNBA	4BNDA	4RGDA	2P14A
3M1XA	4MAKB	4X9TA	4B8XB	3N6YA	4PW0A	5ANPB	1VYRA	3ITFB
1UFYA	2A6ZA	3GJYA	4NDOA	1QQFA	1Y43B	3RPDB	4CNGB	3H7HB
3HX8B	2CVIB	3RL5A	5LALB	3MXZA	1GQIB	4IL7A	5CTVA	3ULJB
3H5JA	1SFSA	3QZMA	4UZ1A	3MD7A	3DFFA	2QJZB	2QPXA	
3U7ZA	4HCSA	2P4HX	2FB6A	5YO6A	2WCWB	2JCBB	5IN4B	
1JFBA	5M1PB	2FCLA	1PKHB	3KB9A	3OOUA	5X89A	2OZHA	
4CE8C	1R6XA	1L9XC	2DDRB	3TVTB	3P0KA	4DQ6B	2EV1B	
2QSKA	3CJSA	3V46A	5Y37A	5NJOA	4W6YA	4F98A	5CWGA	
2W39A	3RQ9B	1YU0A	2XU3A	4A6QA	5GWNA	2DS5A	4ZA9A	
3W61A	1NLQB	2HOXD	3UP3A	2XDWA	5OBTF	2JFRA	2OIZD	
3CIMA	6GAJC	4ANNA	2ET1A	4I6XA	3T2CA	1RYQA	4QASB	
2B4HB	4QC6A	1AHOA	5AJGA	3LYEA	5E1WA	3CHJA	2VY8A	
4Y7LA	4ZVFA	1WDDS	5MUJA	1Y9LA	4G78A	4ERCA	4RLCA	
5EWUA	4ZW9A	4G7XB	4LIXA	1SX5B	3FCNA	1X8QA	2OZJA	
1JYKA	3QU3C	4YTWD	4EADA	3NRFB	3RM3A	4KQDB	3SY1A	
4X33B	4ATEA	5E50A	1D8WD	5O45A	4MTUA	1JI7C	4WUTA	
4RK4A	4FS7A	5XDTA	3O9ZA	3AWUA	4D6GA	1YLXA	4G4KB	
2F5TX	4HBQA	4X33A	3EO6B	1GXUA	3ALJA	3HLXD	3NYTA	
5FD9A	5GI7A	2QF4A	2CVEA	5YHRA	2VK8C	5WGIA	1VH5A	
4YKIB	5HBPA	3LKMA	4DB5A	4EU9A	4PHRA	4W64A	5MULA	

Table S2. The training set of COMTOP.

1A3CA	1RYOA	2CZLA	2QNGA	3BUUA	3LHNA	3VZ9B	4GXWA	4QHQA
1A62A	1RYQA	2D3DA	2QRLA	3BWHA	3LKMA	3W07A	4GZCA	4QKDA
1BGFA	1S3CA	2D5MA	2QSWA	3BWZA	3LLOA	3W42A	4H7WA	4QLPB
1BKRA	1S9UA	2DDXA	2QZCA	3BY8A	3LTIA	3W61A	4H8EA	4QXLA

1C1DA	1S29A	2DKOA	2R0XA	3CECA	3LWXA	3WCQA	4HC9A	4QY7A
1C7KA	1SAUA	2DKOB	2R2ZA	3CHJA	3LYEA	3WDCA	4HCSA	4R1JA
1CC8A	1SFSA	2DSKA	2R5OA	3CHMA	3M1XA	3WDNA	4HE6A	4R2XB
1CY5A	1SZ7A	2DTJA	2R9FA	3CI3A	3M5QA	3WH1A	4HI8B	4R9PA
1DCSA	1T3YA	2DXAA	2R16A	3CIMA	3M9QA	3WH2A	4HLYA	4RAYA
1DD9A	1T5BB	2DXUA	2R31A	3CJSB	3M66A	3WIWA	4HNOA	4RCJA
1DG6A	1T8KA	2DY0A	2RA9A	3CKKA	3MAOA	3WJTA	4HS1A	4REIA
1DJ0A	1T92A	2E3HA	2RDQA	3CLMA	3MBRX	3WURA	4HTGA	4REOA
1DK8A	1TBFA	2E3NA	2RE2A	3COVA	3ME7A	3WZ3A	4HU2A	4RLCA
1DS1A	1TP6A	2EGVA	2RFRA	3CT5A	3MEAA	3X2MA	4I0WA	4RPMA
1E7LA	1TT8A	2ERFA	2RHFA	3D0JA	3MQZA	3X34A	4I6XA	4RRIA
1E58A	1TUAA	2ET1A	2RILA	3D1KA	3MU7A	3ZDBA	4I66A	4RU3A
1EB6A	1U7IA	2EW0A	2RKQA	3D3BA	3MVCA	3ZHIA	4I71A	4RVQA
1EJ8A	1U84A	2F5TX	2TPSA	3D3BJ	3MWXA	3ZHOA	4IAUA	4RWUA
1EKQA	1UCDA	2F9HA	2UU8A	3D4EA	3MWZA	3ZJAA	4IC4A	4RZ9A
1EUVA	1UCSA	2F23A	2V1MA	3D06A	3MXZA	3ZOJA	4IGIA	4S39A
1EUWA	1UFYA	2F60K	2V3GA	3D9NA	3MYXA	3ZSJA	4IILA	4TKBA
1F9VA	1UI0A	2F62A	2V4XA	3DB7A	3N2WC	3ZUCA	4IIYA	4TKCA
1F86A	1UOYA	2FCJA	2V7FA	3DFFA	3N08B	3ZYPA	4IN0A	4TSDB
1FCYA	1USMA	2FCLA	2V8FA	3DFGA	3N10A	3ZZSG	4INWA	4TTWA
1FM0E	1UUYA	2FCOA	2V9LA	3DGTA	3NBCA	4A02A	4IPUA	4TVOA
1FO8A	1V05A	2FCWB	2V9VA	3DKMA	3NO0A	4A2VA	4IQBA	4TVVA
1FP2A	1V5IB	2FGQX	2VB1A	3DNJA	3NUFA	4A3PA	4J5RB	4TXRA
1FYEA	1V7ZA	2FKKA	2VLQA	3DO8A	3NVWB	4A4JA	4J6OA	4U0OB
1G2RA	1VBWA	2FP1A	2VNGA	3DQYA	3O2RA	4A6QA	4J7NA	4U9HS
1G6XA	1VD6A	2FUPA	2VOKA	3DXLA	3O7BA	4A7UA	4J7QA	4U89A
1G61A	1VE4A	2G3RA	2VPAA	3E8TA	3O12A	4A29A	4J8SA	4UE8A
1GK9A	1VH5A	2G84A	2VWSA	3EDHA	3OBLA	4ABLA	4JBDA	4UHQA
1GMUA	1VK1A	2GB4B	2VXNA	3EERA	3OBQA	4AC1X	4JF8A	4UOBA
1GP0A	1VKKA	2GGCA	2VZCA	3EF8A	3OCUA	4ACJA	4JG2A	4UQWB
1GPPA	1VL7A	2GKEA	2W1RA	3EJFA	3OE3A	4AF8A	4JGIA	4UYBA
1GV9A	1VLYA	2GQTA	2W3QA	3EJVA	3OGNA	4AFFA	4JHTA	4V0SB
1GVPA	1VMHA	2GS5A	2W15A	3ELFA	3ORUA	4ALZA	4JJAA	4V3IA
1GWMA	1W0HA	2GUIA	2W31A	3EN0A	3OYVA	4ATEA	4JP6A	4V33A
1GXUA	1W0NA	2GZSA	2W39A	3ER7A	3P02A	4AU1A	4JTMA	4W8HA
1GYXA	1W4SA	2H1VA	2W47A	3EYEA	3P3CA	4AY0A	4K12A	4W8PA
1H32A	1W66A	2H8EA	2W50A	3F2ZA	3P4HA	4B0ZA	4KEFA	4W8QA
1H99A	1WCWA	2HHCA	2WDSA	3F14A	3PD7A	4B1YB	4KL0A	4W64A
1I1WA	1WERA	2HIYA	2WFIA	3F43A	3PHXA	4B5OA	4KQIA	4WBJB
1I71A	1WHIA	2HUHA	2WFWA	3FDEA	3PJPA	4B21A	4KRUA	4WIQA
1IFRA	1WHZA	2HW2A	2WJ5A	3FGYA	3PLUA	4B89A	4KT3A	4WJTA
1IOMA	1WN2A	2HX0A	2WK1A	3FN5A	3PVHA	4BFOA	4KU0D	4WKAA
1IQZA	1WPNB	2HX5A	2WNFA	3FRHA	3Q6BA	4BGBA	4L2IA	4WP9A
1ISUA	1WTJB	2I5UA	2WNKA	3FSOA	3Q46A	4BGCA	4L2IB	4WPKA
1J3AA	1X0TA	2I53A	2WNPF	3FSSA	3Q64A	4BJ0A	4L9PA	4WQKA
1J77A	1X6OA	2IA1A	2WQFA	3FTDA	3QL9A	4BJIA	4L57A	4WSFA
1J98A	1X6ZA	2II2A	2WTPA	3FWKA	3QNSA	4BK7A	4LJOA	4WUIA
1JF4A	1X8QA	2IMFA	2WY4A	3FYMA	3QP4A	4BN4A	4LRUA	4WWFA
1JHJA	1X91A	2IMQX	2WZOA	3G0KA	3QPAA	4BNDA	4LTTA	4WZXA
1JL1A	1XBIA	2IUWA	2X2SA	3G46A	3QX1A	4BOQA	4M8AA	4X2RA
1JM1A	1XD3A	2IXMA	2X5NA	3G91A	3QZBA	4BOUA	4M91A	4X7GA

1JOVA	1XLQA	2J6VB	2X5XA	3GA4A	3QZMA	4BPFA	4MAIA	4X9RA
1K3XA	1XMTA	2J8KA	2X5YA	3GAEA	3QZRA	4BPSA	4MC3A	4X9XA
1K4IA	1XSZA	2J43A	2X9ZA	3GDMA	3QZXA	4BQYA	4MNCA	4X33A
1K4NA	1XUBA	2JCBB	2X49A	3GE3E	3R72A	4BT7A	4MNOA	4XDUA
1K5CA	1Y6XA	2JE6A	2XIOA	3GHAA	3R87A	4BU0A	4MTMA	4XDXA
1K7CA	1Y8AA	2JE6I	2XJPA	3GKJA	3RL5A	4BYZA	4MTUA	4XEZA
1KGDA	1Y43B	2JEKA	2XN6A	3GKRA	3RLGA	4C6AA	4MUQA	4XINA
1KNMA	1YD0A	2JFRA	2XODA	3GNZP	3ROFA	4CBUG	4MVKA	4XJ5A
1KOEA	1YG9A	2JLIA	2XOMA	3GOEA	3RPZA	4CD8A	4MYKA	4XMRB
1KQ6A	1YU0A	2MCMA	2XU3A	3GP6A	3RT2A	4CFIA	4MZCA	4XPXA
1KQFC	1Z2UA	2NLRA	2XW6C	3GWIA	3RX9A	4CHIA	4N1IA	4XTBA
1KT6A	1Z3EB	2NN5A	2XWVA	3GXHA	3RZNA	4CNGB	4N13A	4XXFA
1KWFA	1Z6NA	2NNUA	2Y0OA	3H0NA	3S2JA	4CNNA	4N67A	4XXXA
1KYFA	1Z67A	2NQWA	2Y8YA	3H4OA	3S4EA	4CO8A	4NI6A	4XZFA
1LQVA	1Z70X	2NR7A	2Y78A	3H5JA	3S9XA	4CVRA	4NNOA	4Y0GA
1LWBA	1Z72B	2NRRA	2YH5A	3H7HB	3SBMA	4D0QA	4NOAA	4Y1WA
1M1FA	1ZCEA	2NSZA	2YKZA	3H7IA	3SD2A	4D7JA	4NZKA	4Y7LA
1M2DA	1ZHVA	2NUHA	2YMVA	3HNYM	3SIGA	4D8BA	4O0AA	4Y9IA
1M4LA	1ZL0A	2NWFA	2YN0A	3HPCX	3SK7A	4DB5A	4O0CA	4Y9WA
1M7GB	1ZMAA	2O0MA	2YVTA	3HWUA	3SO6A	4DDPA	4O1RA	4Y88A
1M15A	1ZZKA	2O2KA	2YZYA	3HZ8A	3SOJA	4DK2A	4OA3A	4YCBB
1MC2A	2A0BA	2O7AA	2Z5WA	3IE4B	3SY1A	4DO4A	4OD6A	4YDRB
1MK0A	2A6ZA	2O8QA	2Z6RA	3IEZA	3SZ3A	4DVCA	4OFAA	4YECA
1MUWA	2AGKA	2O90A	2Z51A	3IP0A	3SZVA	4E4RA	4OH7A	4YECB
1N62A	2AP3A	2OB3A	2ZFDB	3IP8A	3T3LA	4EGUA	4OI3A	4YEPA
1N62C	2ASBA	2OB5A	2ZHJA	3IPJA	3T7LA	4EICA	4OJXA	4YGBD
1NC5A	2B0AA	2OHWA	2ZNRA	3IT3A	3T92A	4EQPA	4OMBA	4YLQT
1NU0A	2B97A	2OKFA	2ZOUA	3IVVA	3TBNA	4ES1A	4OQPA	4YMYA
1NUYA	2BBRA	2OLMA	2ZPTX	3IX3A	3TG2A	4ESMA	4OUSA	4YQDA
1NXMA	2BKXA	2ORWA	2ZS0C	3JQ0A	3TKFA	4F01A	4P3VA	4YSIA
1O7IA	2BMOB	2OV0A	3A2ZA	3JTZA	3TM8A	4F1VA	4P5NB	4YTKA
1O7JC	2BOGX	2OVJA	3A35A	3JUDA	3TOWA	4F2EA	4P7OA	4YWKA
1OCYA	2BU3A	2OXGD	3ACXA	3JUMA	3TUTA	4F98A	4P7XA	4Z0YB
1OD3A	2BWRA	2OZJA	3AGNA	3JYOA	3TYSA	4FK9A	4PDNA	4Z7EB
1ODMA	2BZ1A	2P0NA	3AJ4A	3K5JA	3U2UA	4FUUA	4PF3A	4Z7XA
1OI0D	2C2UA	2P5KA	3AJ6B	3K7PA	3U7ZA	4FZPA	4PHRA	4Z47A
1OK0A	2C3VA	2P6WA	3AJDA	3KB9A	3U25B	4G0XA	4PI8A	4ZAVA
1OQJA	2CB8A	2P9WA	3AKSA	3KFFA	3U65B	4G6TA	4PIOA	4ZC3A
1P5ZB	2CC6A	2P51A	3AMRA	3KGKA	3UB6A	4G7XB	4PLZA	4ZCEB
1PKHB	2CCQA	2PIEA	3AWUA	3KM5A	3UI4A	4G9SA	4PNOA	4ZGFA
1PZ4A	2CCVA	2POFA	3AWUB	3KWRA	3UIDA	4G9SB	4PQDA	4ZHWA
1QQFA	2CDPA	2PQ7A	3B64A	3KYJA	3ULTA	4G54A	4PQQA	4ZILB
1QW2A	2CI1A	2PQ8A	3B79A	3KYZA	3UP3A	4G78A	4PS6A	4ZJHA
1QWGA	2CIUA	2Q5CA	3BA3A	3L46A	3UR8B	4GB5A	4PUXA	4ZOTA
1R6XA	2CIWA	2Q6KA	3BEDA	3L51B	3URRA	4GC3A	4PZ3A	4ZR8B
1R7JA	2CKKA	2QCPX	3BEXA	3L81A	3V46A	4GEIA	4Q2LA	4ZY9B
1R45A	2CVEA	2QF4A	3BGUA	3LAGA	3VGIA	4GJZA	4Q4W2	5A0DD
1ROCA	2CWSA	2QFEA	3BHWA	3LAXA	3VJ9A	4GMQA	4Q4W3	
1RTTA	2CXAA	2QIPA	3BM7A	3LD7A	3VQJA	4GS3A	4QA8A	
1RV9A	2CXYA	2QJLA	3BS4A	3LGBB	3VUBA	4GT9A	4QBOA	
1RXIA	2CYJA	2QL8A	3BT5A	3LHCA	3VVVA	4GWBA	4QGOA	

Table S3. 239 non-redundant proteins used for testing.

5A0RA	5C9OA	5E50A	5H3VA	5JEEA	5LUND	5OHQA	5V1YB	
5A1IA	5C12A	5EHIC	5H6XB	5JH8A	5LZKB	5OL4B	5V3NA	
5A3AA	5C33B	5EJXA	5H7EA	5JI7A	5LZNA	5OL9A	5V6JC	5Y2SA
5A6WB	5C98A	5EL9A	5HB6A	5JICA	5M0WA	5OMTA	5V44A	5Y9AA
5A67A	5CD2A	5EMIA	5HDKD	5JIGA	5M0YB	5OPZA	5VGBA	5YDEA
5AGIA	5CDKA	5EQ0A	5HEEA	5JJ2A	5M1PB	5OVOA	5VGLA	5YGBA
5AGRA	5CEGB	5EQ7A	5HHJA	5JO8A	5M17A	5SV2A	5VHGA	5YO6A
5AJGA	5CGQA	5EWUA	5HJ9A	5JVIE	5MAWE	5SV5A	5VX1B	5YQJA
5AMHA	5CKLA	5EWYA	5HQHA	5K87A	5MPWC	5SY4B	5VYQA	5Z42A
5ANPB	5CL8A	5F6RB	5HTLA	5K91A	5MUJA	5T1IB	5W2FA	5ZBYA
5APGB	5CR4B	5FAFA	5I0YA	5KLAA	5MY5A	5T5LA	5W2IA	6AMGB
5AQ0A	5CTVA	5FBFA	5ICUA	5KO5A	5NCGB	5TC6A	5W83A	6AO9A
5AZWA	5CWHA	5FISA	5IDBA	5KO9A	5NCJB	5TCBA	5W98A	6B1KA
5AZXB	5D1MB	5FJDC	5IDHA	5KTNA	5NJOA	5TDAA	5WGIA	6B2VA
5B1AP	5D2KA	5FOTA	5IG6A	5KVBA	5NOAA	5TJZA	5WK0A	6B9HA
5B1AS	5D4VB	5FSVA	5IHWA	5KXHA	5NQOA	5TKWA	5WLFA	6B9XB
5B1RA	5D7UB	5FU5A	5IO9B	5KZZA	5NRMA	5TLEA	5WUCA	6B9XC
5B4BB	5D8VA	5FVND	5IUCB	5L0VA	5NSAA	5TSQA	5X5MA	6BCBA
5B08A	5DBLA	5FYPB	5IWHA	5L37C	5NVGA	5U81A	5X9IB	6CD9A
5B8DA	5DFSA	5G3YA	5IXHB	5L74A	5NVIA	5UE1B	5X9LA	6COJB
5BPKA	5DHDA	5GGNA	5J3TA	5LALB	5NW3A	5UEBB	5X57A	6EIOA
5BPKC	5DICA	5GJIA	5J4FA	5LBDB	5O5SA	5UFYA	5X89A	6ENSA
5BY5A	5DLED	5GMDA	5J4LA	5LEOA	5O6TA	5UGRA	5XBIB	6EOZA
5BYKA	5DMAA	5GNGB	5J6YA	5LJPA	5O8MD	5UUKA	5XDTA	6ETLA
5C5GA	5DMDB	5GS7A	5J80A	5LP9A	5O9MA	5UWZA	5XJ5A	6FNUA
5C5ZB	5DZEA	5GV0A	5JBNB	5LS7D	5O15A	5UX1C	5XK6B	
5C6SA	5E4BB	5H0MA	5JDAA	5LU5D	5O75A	5V01A	5XM5B	

Table S4. 30 CASP13 target domains used for testing.

T0950-D1	T0957s2-D1	T0951-D1	T1009-D1	T1009-D1	T0960-D1
T0953s1-D1	T0960-D2	T0960-D5	T1011-D1	T1011-D1	T0960-D4
T0953s2-D2	T0963-D2	T0963-D5	T0953s2-D1	T0953s2-D1	T0963-D1
T0953s2-D3	T0968s1-D1	T1003-D1	T0958-D1	T0958-D1	T0963-D4
T0957s1-D1	T0968s2-D1	T1016-D1	T1005-D1	T1005-D1	T1011-D2

Table S5. 25 CASP14 target domains used for testing.

T1027-D1	T1037-D1	T1042-D1	T1047s2-D1	T1065s1-D1
T1029-D1	T1038-D2	T1043-D1	T1047s2-D3	T1065s2-D1
T1031-D1	T1039-D1	T1046s1-D1	T1049-D1	T1074-D1
T1033-D1	T1040-D1	T1046s2-D1	T1053-D1	T1082-D1
T1035-D1	T1041-D1	T1047s1-D1	T1053-D2	T1090-D1

Table S6. 57 non-redundant TM proteins used for testing.

2K9PA	2NDJA	5OQKA	5ZAZA	6CFWI	6VK0D
2KSDA	3NYMA	5VKVA	6A69B	6CFWM	6WB91
2KSEA	4QNDA	5XEFA	6B2ZT	6F0KA	6WB94
2KSFA	5EDLA	5XTDE	6CFWA	6ND1E	6WB95
2LOMA	5KTFA	5XTDF	6CFWB	6S3KA	6WB96
2LORA	5KYHA	5XTDI	6CFWC	6S7TC	6WW7E

2M0QA	5LNKY	5XTDL	6CFWD	6S7TE	6WW7F
2MFRA	5LNKZ	5XTDN	6CFWE	6S7TF	
2MMUA	5MG3F	5XTDO	6CFWF	6S7TG	
2N4XA	5OC0A	5YQ7C	6CFWG	6UKJA	

Table S7. Parameters 〖λ(m1)〗_i (for the seven method COMTOP) obtain from the overall training process are shown in column 1 to 6 respectively.

**Methods**	**Weight value**					
	**Training-model_5L** $\boldsymbol{\lambda(m1)}$	**Training-model_3L** $\boldsymbol{\lambda(m1)}$	**Training-model_2L** $\boldsymbol{\lambda(m1)}$	**Training-model_L** $\boldsymbol{\lambda(m1)}$	**Training-model_L/2** $\boldsymbol{\lambda(m1)}$	**Training-model_L/5** $\boldsymbol{\lambda(m1)}$
CCMpred	0.005	0.117	0.085	0.077	0.00001	0.233
DeepCOV	0.433	0.433	0.357	0.356	0.440	0.292
EVfold	0.008	0.000002	0.001	0.108	0.000006	0.005
NNcon	0.0003	0.004	0.082	0.021	0.000001	0.004
plmDCA	0.008	0.000006	0.055	0.063	0.0005	0.008
PconsC4	0.528	0.445	0.408	0.364	0.546	0.446
PSICOV	0.016	0.000003	0.002	0.009	0.000083	0.000083
